# Enhanced Human Antigen‐Specific B Cell Responses Using In Vitro 3D Tonsil Cultures Containing Stromal Cells

**DOI:** 10.1002/adhm.202504886

**Published:** 2026-04-16

**Authors:** Maaike V. J. Braham, Marlon de Gast, Liubov Babii, Sabine Kruijer, Theo M. Bestebroer, Mathilde Richard, Mathieu Claireaux, S. Marieke van Ham, Jelle de Wit, Cécile A. C. M. van Els, Anja ten Brinke, Marit J. van Gils

**Affiliations:** ^1^ Sanquin Amsterdam The Netherlands; ^2^ Amsterdam Institute for Immunology and Infectious Diseases Amsterdam The Netherlands; ^3^ Centre For Immunology of Infectious Diseases and Vaccines Department of Immune Mechanisms National Institute for Public Health and the Environment Bilthoven The Netherlands; ^4^ Department of Medical Microbiology and Infection Prevention Amsterdam UMC University of Amsterdam Amsterdam The Netherlands; ^5^ Department of Viroscience Erasmus University Medical Center Rotterdam The Netherlands; ^6^ Swammerdam Institute for Life Sciences University of Amsterdam Amsterdam The Netherlands; ^7^ Infectious Diseases & Immunology Department of Biomolecular Health Sciences Faculty of Veterinary Medicine Utrecht University Utrecht The Netherlands

**Keywords:** 3D culture system, antibody‐secreting cells, fibroblastic reticular cells, germinal center response, hydrogel, organoid, vaccine

## Abstract

Germinal centers (GCs) are specialized sites within secondary lymphoid organs where B cells expand, are selected, and mature to produce high‐quality antibodies. Their structural complexity makes them difficult to model in vitro. Here, we developed a human 3D lymphoid culture system combining lymphoid and stromal cells to better mimic GC environments than conventional 2D cultures. Tonsil cells were cultured with or without fibroblastic reticular cells (FRCs) in 2D or 3D hydrogels and stimulated with viral antigens or vaccines. FRC‐supported 3D cultures significantly improved B and T cell survival and promoted reaggregation into follicle‐like structures with. 3D FRC‐supported co‐cultures higher levels of antigen‐specific antibodies, increased frequencies of S‐ or HA‐specific B cells, and enhanced differentiation into antibody‐secreting cells. Importantly, these cultures also showed reduced cell death and lower bystander activation and CXCR4 and CXCR5 expression on CD27^+^CD38^+^ B cells indicated GC‐like polarization. Autologous and allogeneic FRCs performed comparably, supporting the scalability of the model for high‐throughput applications. This 3D platform offers a more physiologically relevant system for studying human GC‐associated immune responses and may facilitate mechanistic research and screening of vaccine immunogens and adjuvants in a controlled laboratory setting.

## Graphical abstract

We developed a fully human 3D tonsil cell culture system incorporating supportive stromal cells that better sustains and activates immune cells than conventional methods. The model generates stronger, more targeted antibody responses to viral antigens and vaccines, providing a physiologically relevant and entirely human platform for studying immune reactions in the lab without the need for animal experiments.

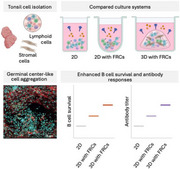

## 1 Introduction

Secondary lymphoid organs (SLOs), such as lymph nodes and tonsils, are highly complex in both structure and cellular composition. Their organization allows immune cells to interact efficiently, supported by stromal networks that guide migration and maintain tissue integrity. Upon antigen exposure, B cell follicles form specialized microanatomical structures called germinal centers (GCs) to generate high‐affinity antibody responses.

A defining feature of GCs is their compartmentalization into dark and light zones. In the dark zone, B cells (CXCR4^hi^CXCR5^+^) proliferate and diversify their receptors through somatic hypermutation (SHM) [1, 2]. In the light zone, B cells (CXCR4^lo^CXCR5^+^) encounter antigen presented by follicular dendritic cells (FDCs) and signals from T follicular helper (Tfh) cells that drive affinity‐based selection of B cells [1, 3, 4]. Together, these processes in the GC generate high‐affinity memory B cells and plasma cells.

While GC organization relies on distinct zones for B cell selection, immune cell positioning within SLOs is further directed by stromal cells, including fibroblastic reticular cells (FRCs) [5, 6, 7]. Once considered merely a structural backbone, FRCs are now recognized as active regulators of lymphocyte migration and activation [8, 9], producing chemokines that guide B and T cell trafficking [10, 11]. By providing structural support and essential chemokines, FRCs are thus critical for the organization and function of GCs [12].

Historically, animal models, particularly mouse models, have played a pivotal role in shaping our understanding of the immune system. While mouse models have yielded valuable insights, interspecies differences in SLO structure, including stromal cell composition and immune cell density, can affect GC formation and function [13]. Furthermore, disparities in antibody repertoires between mice and humans [13, 14] may alter vaccine‐induced immune responses. Consequently, investigating human antigen‐specific immune responses, particularly in the context of vaccine development, necessitates models that recapitulate essential cell types and structures of native human tissues to faithfully replicate GC‐like responses.

To better elucidate human antigen‐specific B cell responses, in vitro models have evolved from simple 2D cultures to increasingly more complex systems that integrate multiple cell types, 3D matrices, and flow. Traditional approaches relied on artificial CD40L stimulation, using either recombinant human CD40L or CD40L‐expressing fibroblasts in combination with various cytokines and B cell activating‐stimuli such as TLR‐ligands [15, 16, 17]. We recently established a synthetic human 3D in vitro lymphoid model capable of inducing B cell differentiation into antibody‐secreting cells [18]; however, this model lacked physiological T cell interactions and provided continuous antigen‐independent CD40L stimulation. To investigate human B and T cell interactions more accurately, including selection and migration/cycling of cells, more advanced models are required. Recent studies combine 3D matrices with organ‐on‐a‐chip technology to generate advanced models, in which B and T cells interact within 3D structures under continuous perfusion [19, 20]. Notably, none of these complex models incorporated a stromal compartment. Additionally, the use of human peripheral blood mononuclear cells (PBMCs) often results in B and T cell populations whose composition and ratios differ from those found in native human SLOs [21].

Recently, alternative in vitro models have been described that use tonsil cells instead of PBMCs. These models have been applied to study human adaptive immune responses to different antigen formats in the context of naturally occurring cellular composition and ratios found in tonsils [22, 23]. These tonsil models, however, are executed in more simplified 2D cultures, therefore lacking a 3D architecture and a stromal compartment, both considered critical factors in SLOs.

A 3D human lymphoid model that incorporates key tonsil‐derived SLO cell populations within a structured 3D environment, could facilitate the study of GC‐like B and T cell responses, including their differentiation, proliferative capacity, and antibody secretion and affinity maturation. In this study, we aimed to advance existing 2D in vitro models by introducing a stromal compartment into a 3D culture setting. Tonsil‐derived cells were used to evaluate the impact of adding FRCs, either autologous or allogeneic. In addition, the use of a PEG‐4MAL‐RGD functionalized matrix on T and B cell responses under various stimulation conditions and experimental set‐ups was evaluated. Our findings show that the 3D model with an integrated stromal compartment enhances B and T cell survival and supports the emergence of GC‐like phenotypic features, as indicated by CXCR4 and CXCR5 expression patterns, and enhances antigen‐specific B cell responses. Collectively, this platform represents a more physiologically relevant in vitro system compared to conventional 2D cultures for investigating human adaptive immune responses and supports further advancements in vaccine development.

## 2 Results

### 2.1 Isolation and Characterization of Lymphoid and Fibroblastic Reticular Cells From Tonsils at Baseline

**FIGURE 1:**
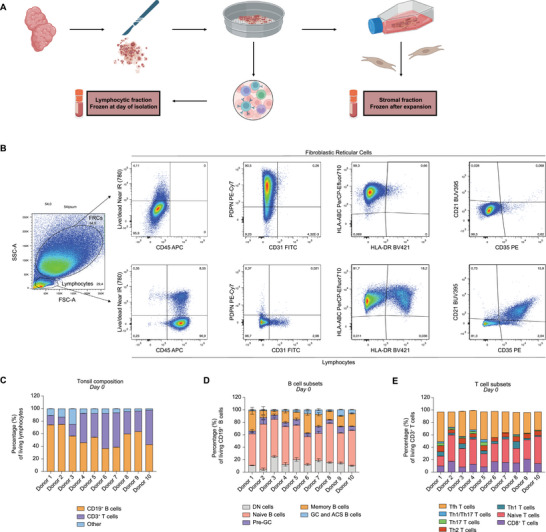
Isolation and characterization of lymphocytes and fibroblastic reticular cell fraction from tonsils. (A) Tonsil cells were isolated from 10 donors following tonsillectomy. Each tonsil was minced into small pieces and sieved. The isolated single‐cell lymphocytic fraction was cryopreserved at day 0. The remaining tissue fragments were cultured for fibroblastic reticular cells (FRCs) isolation. (B) Flow cytometry plots of both the FRC and lymphocyte fractions (both after cryopreservation). FRCs can be characterized as CD45^−^PDPN^+^CD31^−^ cells expressing HLA‐ABC but not HLA‐DR, CD21, and CD35. FRCs and lymphocytes can also be distinguished based on size and forward scatter. (C) Tonsil composition of each donor, expressed in percentage (%) of living lymphocytes, containing mainly CD19^+^ B cells and CD3^+^ T cells in varying ratios. (D) B cell subsets as a percentage (%) of living CD19^+^ B cells, containing double‐negative cells (DN; CD27^−^CD38^−^IgD^−^), naive B cells (CD27^−^CD38^−^IgD^+^), memory B cells (CD27^+^CD38^−^), pre‐GC (CD27^−^CD38^+^), and GC and antibody‐secreting cells (GC and ASC; CD27^+^CD38^+^) in varying ratios per donor. (E) T cell subsets as a percentage (%) of living CD3^+^ T cells, containing CD8^+^ T cells, naive CD4^+^ T cells (CD45RA^+^), Th1 T cells (CD4^+^CD45RA^−^CXCR5^−^CXCR3^+^CCR6^−^), Th2 T cells (CD4^+^CD45RA^−^CXCR5^−^CXCR3^−^CCR6^−^), Th17 T cells (CD4^+^CD45RA^−^CXCR5^−^CXCR3^−^CCR6^+^), Th1/17 T cells (CD4^+^CD45RA^−^CXCR5^−^CXCR3^+^CCR6^+^) and Tfh T cells (CD4^+^CD45RA^−^CXCR5^+^) in varying ratios per donor. Data showing the mean ± SD of technical triplicates.

Both lymphoid (n = 10) and stromal cell (n = 5) fractions were isolated from tonsil donors to study the GC‐like immune responses within their stromal microenvironment (Figure 1A). The processed lymphoid fractions were directly frozen upon isolation, while the stromal fractions were first expanded. The obtained expanded stromal cell fraction was identified as fibroblastic reticular cells (FRCs; CD45^−^PDPN^+^CD31^−^ cells), expressing HLA‐ABC but not HLA‐DR, CD21, and CD35 (Figure 1B) [24].

The lymphocytic compartment, analyzed at baseline (day 0) after freezing, consisted predominantly of CD19^+^ B cells and CD3^+^ T cells in varying ratios, with minor populations of other immune cells present (Figure 1C; Figure S1A). Further analysis of the B cell subsets revealed distinct populations, including naive B cells (CD27^−^CD38^−^IgD^+^), double‐negative (DN; CD27^−^CD38^−^IgD^−^), pre‐GC (CD27^−^CD38^+^), memory B cells (CD27^+^CD38^−^), and a CD27^+^CD38^+^ compartment containing both GC‐like B cells (CD38^+^CD20^+^) and antibody‐secreting cells (ASC; CD38^++^CD20^−^; Figure 1D; Figure S5). Some donors had a mainly naive B cell compartment (up to 75% of B cells), while others had a clear memory B cell compartment (up to 37%). Similarly, T cell analysis demonstrated the presence of various T cell subsets, including Th1, Th2, Th17, and a substantial portion of the GC‐essential Tfh cells, which constituted up to 50% of the total T cells (Figure 1E; Figure S1B).

**FIGURE 2:**
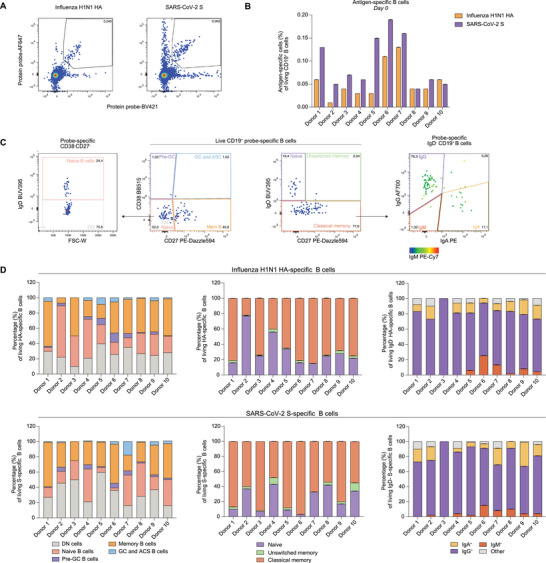
Baseline characterization of influenza H1N1 hemagglutinin (HA)‐specific B cells and SARS‐CoV‐2 wild type (WT) spike‐specific B cells (A) Flow cytometry plots of protein‐specific B cells gating. Viable CD19^+^ B cells were considered protein‐specific when double‐positive for the used protein probes in two fluorescent channels. (B) Influenza H1N1 HA‐specific B cells and SARS‐CoV‐2 WT spike‐specific B cells as a percentage (%) of living CD19^+^ B cells per donor. (C) Flow cytometry plots illustrating the gating strategy for live CD19^+^ probe‐specific B cells. CD27 and CD38 expression (middle left plot) was used to identify naïve/double‐negative (DN; CD27^−^CD38^−^), memory (CD27^+^CD38^−^), pre‐germinal center (pre‐GC; CD27^−^CD38^+^), and germinal center/antibody‐secreting cells (GC/ASC; CD27^+^CD38^+^). From the CD27^−^CD38^−^ subset, DN and naïve B cells were further distinguished based on IgD expression (left plot). From the live CD19^+^ B cell population, IgD and CD27 expression (middle right plot) was used to identify naïve (IgD^+^CD27^−^), unswitched memory (IgD^+^CD27^+^), and class‐switched memory (IgD^−^CD27^+^) B cells. Class‐switched memory B cells were subsequently analyzed for isotype expression and subdivided into IgM^+^, IgG^+^, or IgA^+^ subsets (right plot). (D) Characterization of viable influenza H1N1 HA‐specific B cells (top row) or viable SARS‐CoV‐2 WT spike‐specific B cells (bottom row) in terms of CD27/CD38 expression (left), IgD/CD27 expression (middle) and IgM/IgG/IgA (right) into the populations elaborated upon (C), shown as a percentage (%) of total viable protein specific B cells. Data showing the mean ± SD.

To study B and T cell responses to SARS‐CoV‐2 virus and influenza H1N1 virus antigens, the presence of antigen‐specific B cells to both viruses were characterized at baseline, focusing on their specificity for SARS‐CoV‐2 Spike (S) and influenza H1N1 hemagglutinin (HA; Figure 2A). Antigen‐specific B cells were detected in all donors for both antigens, at varying frequencies and phenotypes (Figure 2B). Antigen‐specific B cells were mainly found in the class‐switched double‐negative (CD27^−^IgD^−^) and classical memory compartment (CD27^+^IgD^−^), mainly being IgG^+^ cells (Figure 2C,D). The observed variability in antigen‐specific B cell populations underscores considerable donor‐to‐donor differences in immune histories and highlights the importance of analyzing baseline cells as the starting point for subsequent in vitro cultures.

### 2.2 Setup of an In Vitro Lymphoid Model, Enhancing Traditional 2D Cultures by Incorporating Both FRCs and a 3D Matrix Resulting in Cultures Resembling SLO Structures

**FIGURE 3:**
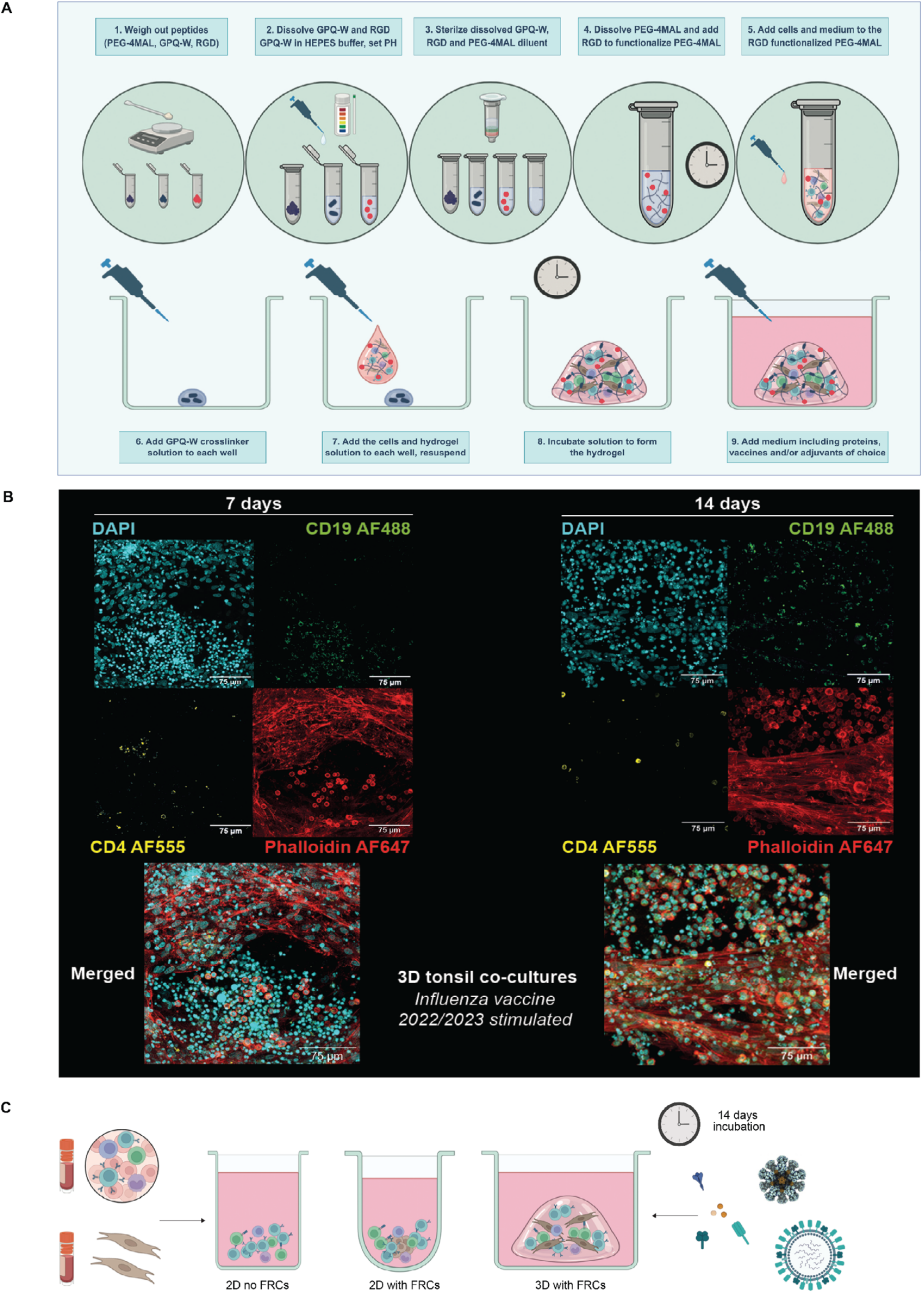
Preparation of 2D and 3D tonsil co‐cultures with and without FRCs and confocal imaging. (A) Schematic overview of the method used to prepare 3D tonsil co‐cultures using a 5% (w/v) 20 kDa PEG‐4MAL, RGD functionalized (GRGDSPC), GPQ‐W crosslinked (GCRDGPQGIWGQDRCG) hydrogel. The PEG‐4MAL macromer, adhesive RGD peptide, and cross‐linker GPQ‐W were weighed out, dissolved, PH adjusted, and filtered. The PEG‐4MAL and RGD solution were combined into a functionalized PEG‐4MAL precursor solution. Cells were added to the precursor solution and then mixed with the cross‐linking peptide solution pipetted in the bottom of each well. After casting and cross‐linking the hydrogel, medium was added. (B) Confocal imaging of 3D tonsil co‐cultures with FRCs. Cyan = DAPI, green = CD19 Alexa fluor 488, yellow = CD4 Alexa fluor 555, red = phalloidin Alexa fluor 647. Left image: influenza vaccine 2022/2023 (Influvac Tetra 2022/2023) stimulated culture on day 7. Scale bars represent 100 µm. Right image: influenza vaccine 2022/2023 stimulated culture on day 14. Scale bars represent 75 µm. (C) Cryopreserved tonsil cells and FRCs were thawed and plated out in the two 2D (left and middle) and 3D (right) tonsil (co‐)culture setups, after which they were studied for their B and T cell responses for up to 14 days using varying antigen exposures.

To establish a culture system that closely mimics SLOs, we aimed to enhance traditional 2D cultures by incorporating FRCs and a 3D matrix. The 3D matrix used is a synthetic PEG‐4MAL hydrogel (5% (w/v)), free of any animal‐derived components, cross‐linked with a protease degradable cross‐linking peptide GCRDGPQGIWGQDRCG (GPQ‐W) peptide and cell‐adhesive peptide GRGDSPC (RGD), which mimics the minimal integrin‐binding motif found in extracellular matrix proteins [18] (Figure 3A). The incorporated RGD peptides promote integrin‐mediated adhesion, allowing cells to attach and spread effectively within the 3D environment, thereby enhancing cellular interactions in a more physiologically relevant manner compared to 2D cultures. Within these hydrogels, the tonsillar lymphocytic and FRC fractions were mixed, forming cultures that were visualized using fluorescent confocal microscopy. Imaging revealed reaggregation and close interactions between B and T cells (Figure 3B). The FRCs form structural niches/follicles within the hydrogel (Figure 3B), providing a supportive environment where B and T cells reside and interact, mimicking the basic microarchitecture of SLOs. Large aggregating lymphocyte structures were observed in stimulated cultures (e.g., Influenza vaccine 2022/2023, Figure 3B), but not in unstimulated cultures (Figure S2A).

Traditional 2D lymphoid cultures were compared to 2D FRC‐supported cultures and 3D FRC‐supported cultures (Figure 3C). Medium optimization was carried out in 2D cultures, which showed the lowest overall baseline cell survival. Various types of medium, with and without cytokines, were tested to determine the optimal baseline culture conditions for both antigen‐ and non‐antigen‐specific cells in all culture set‐ups (Figure S2B–E). Accordingly, 3D cultures were de‐crosslinked to recover cells for flow cytometric evaluation. 2D cultures showed low survival of B cells without FRCs (Figure S2B), which was not improved through the addition of cytokines IL4 and IL21 (Figure S2C), but instead caused extensive expansion in the CD8^+^ T cell compartment (Figure S2D). Interestingly, when FRCs were added, the effect of IL4 and IL21 on CD8^+^ T cells was dampened. Even in the absence of stimulation, FRCs significantly improved lymphocyte survival in 2D cultures (*p* < 0.0001), with further improvements observed when FRCs were combined with a 3D matrix (*p* < 0.0001; Figure S2E). This effect was independent of recombinant human B cell activating factor (BAFF) supplementation (Figure S2E), which was therefore excluded from subsequent cultures. IMDM alone, without additional cytokines, was sufficient to support optimal lymphocyte survival across all culture conditions and was thus selected for further experiments.

### 2.3 FRC‐Supported Cultures Improve Lymphocyte Viability, Differentiation, and Antibody Production After Antigen Stimulation

Responses of 2D cultures, 2D FRC‐supported cultures, and 3D FRC‐supported cultures were evaluated when stimulated with various antigen formats (Figure 3C). These included SARS‐CoV‐2 WT S or influenza H1N1 HA recombinant proteins, both with and without the adjuvant R848 (TLR7/8‐agonist), SARS‐CoV‐2 WT S nanoparticles (NPs), and two types of influenza vaccines. This setup aimed to mimic conditions in SLOs, where GCs form in response to antigen or vaccine stimulation.

**FIGURE 4:**
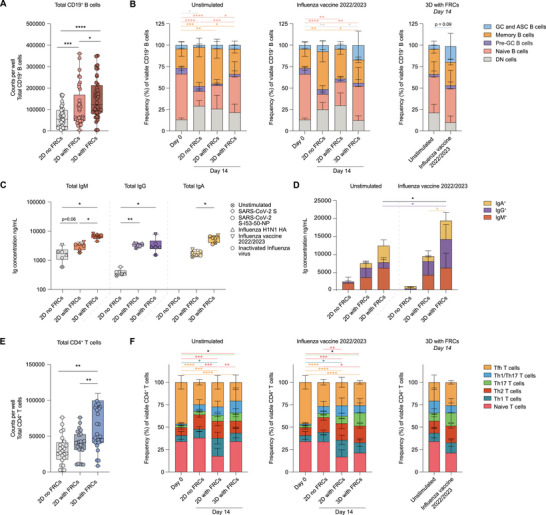
Impact of FRCs and 3D culture on B and T cell survival, differentiation, and immunoglobulin production in response to viral antigens. Survival of B and T cells when cultured in 2D without FRCs, 2D with autologous FRCs, or 3D with autologous FRCs, either left unstimulated or stimulated with influenza vaccine 2022/2023 (Influvac Tetra 2022/2023) (A) Total counts of viable CD19^+^ B cells per well at day 14. Each dot represents an individual donor for one stimulation condition. All stimulation conditions are shown pooled (excluding R848‐adjuvanted conditions). Data are presented as median with interquartile range; whiskers indicate min to max. (B) Percentage of CD19+ B cell subsets, including double‐negative cells (DN; CD27^−^CD38^−^IgD^−^), naive B cells (CD27^−^CD38^−^IgD^+^), memory B cells (CD27^+^CD38^−^), pre‐GC (CD27^−^CD38^+^), and GC and antibody‐secreting cells (GC and ASC; CD27^+^CD38^+^). The left panel shows unstimulated conditions, the middle panel influenza vaccine–stimulated conditions, and the right panel a direct comparison of unstimulated vs. influenza vaccine in 3D FRC‐supported cultures. (C) Concentrations of total IgM, IgG, and IgA (from left to right) in available supernatants collected at day 14, quantified by ELISA. Each dot represents the median of n = 5 tonsil donors for one stimulation condition, with different symbols denoting stimulation conditions. All stimulation conditions are included except R848‐adjuvanted conditions. Ig concentration is in ng/mL. (D) Concentrations of IgM, IgG, and IgA in supernatants after 14 days, either unstimulated or stimulated with influenza vaccine. (E) Total counts of viable CD4^+^ T cells per well at day 14. Each dot represents an individual donor for one stimulation condition. All stimulation conditions are shown pooled (excluding R848‐adjuvanted conditions). Data are presented as median with interquartile range; whiskers indicate min to max. (F) Frequency of naive CD4^+^ T cells (CD45RA^+^), Th1 T cells (CD4^+^CD45RA^−^CXCR5^−^CXCR3^+^CCR6^−^), Th2 T cells (CD4^+^CD45RA^−^CXCR5^−^CXCR3^−^CCR6^−^), Th17 T cells (CD4^+^CD45RA^−^CXCR5^−^CXCR3^−^CCR6^+^), Th1/17 T cells (CD4^+^CD45RA^−^CXCR5^−^CXCR3^+^CCR6^+^), and Tfh T cells (CD4^+^CD45RA^−^CXCR5^+^). Baseline (day 0) composition is compared with day 14 cultures. The left panel shows unstimulated conditions, the middle panel influenza vaccine–stimulated conditions, and the right panel a direct comparison of unstimulated vs. influenza vaccine in 3D FRC‐supported cultures at day 14. Panels A, C, and E were analyzed using paired non‐parametric tests (Wilcoxon signed‐rank). Panels B, D, and F were analyzed using paired tests with correction for multiple comparisons (Benjamini–Hochberg). Data represent mean ± SD of *n* = 5 tonsil donors. ^*^ = *p* < 0.05, ^**^ = *p* < 0.01, ^***^ = *p* < 0.001, ^****^ = *p* < 0.0001; statistical significance is indicated in the color of the corresponding experimental group with a black line depicting differences between total amounts.

Influenza vaccines induced the most consistent donor‐wide responses and are therefore highlighted (Figure 4); results for the other antigens are presented in Figure S3. For the CD19^+^ B cell analyses, and for the overall analyses of total antibodies and total T cells, R848‐adjuvanted conditions were excluded because this stimulus induced strong and (at least partly) non‐specific activation (Figures S3 and S5G–J), overpowering antigen‐specific B and T cell responses with broad overall cellular activation independent of antigen recognition. At day 14, cultures with FRC support contained significantly more viable CD19^+^ B cells than 2D lymphoid cultures without FRCs (2D with FRCs, *p* = 0.003; 3D with FRCs, *p* = 0.001), with the highest number of viable cells in the 3D condition (Figure 4A; Figures S3A and S4). For the CD19^+^ B cell analyses, and for the overall analyses of total antibodies and total T cells, R848‐adjuvanted conditions were excluded because this stimulus induces strong non‐specific activation (Figure S3).

Culturing alone reshaped B cell composition over 14 days, shifting from high frequencies of naïve cells to higher proportions of other subsets (Figure 4B; Figure S3C). The 3D cultures with FRCs contained the largest naive compartment at day 14, indicating that this condition supports naïve B cell survival (Figure S3C). In addition, GC and ASC populations were more prominent in 3D FRC‐supported cultures than in 2D cultures, particularly after stimulation with the influenza vaccine, which showed a trend toward higher GC/ASC frequencies compared to unstimulated conditions (4‐fold increase; *p* = 0.09; Figure 4B). In contrast, stimulation with the general activator R848 produced a distinct B cell composition, with even higher GC/ASC frequencies in 3D cultures with FRCs compared to unstimulated conditions (*p* = 0.03; Figure S3C).

Consistent with enhanced B cell survival and differentiation, total antibody production was higher in FRC‐supported cultures, especially in 3D (Figure 4C,D). Total IgM secretion was significantly higher in 3D cultures with FRCs than in either 2D condition (*p* = 0.03 for both), with 2D cultures showing only a trend toward higher levels in the presence of FRCs compared to those without (*p* = 0.06; Figure 4C). Total IgG levels were elevated in both FRC‐supported conditions compared to 2D cultures without FRCs (*p* = 0.03 for both; Figure 4C), while total IgA secretion was specifically enhanced in 3D cultures with FRCs compared to 2D FRCs (*p* = 0.03). Following influenza vaccine stimulation, antibody secretion increased further in 3D cultures with FRCs compared to their unstimulated condition (*p* = 0.02), an effect largely driven by higher IgG levels (*p* = 0.03; Figure 4D). R848 stimulation, irrespective of whether combined with spike or HA, strongly enhanced antibody production (Figure S3D). In 3D FRC‐supported cultures, R848 significantly increased total IgM, IgG, and IgA secretion (all *p* < 0.0001), while in 2D FRC cultures it selectively boosted IgM levels (*p* < 0.0001).

T cell analysis revealed that FRC support improved CD4^+^ T cell recovery as well, with significantly higher counts in 3D cultures with FRCs compared to 2D cultures without FRCs at day 14 (*p* = 0.003; Figure 4E; Figure S3B). Culturing reshaped CD4^+^ T cell composition, with a significant decrease in naive T cells in FRC‐supported conditions stimulated with influenza vaccine (2D with FRCs, *p* = 0.002; 3D with FRCs, *p* = 0.03; Figure 4F), accompanied by a relative increase in effector subsets including Th1, Th2, Th17, and Th1/17 (Figure 4F).

Together, these results show that 3D FRC co‐cultures provide a supportive niche for both lymphocyte survival and differentiation.

### 2.4 3D FRC‐Supported Cultures Show Amplified Specific B Cell Responses to Varying Antigen Stimulations, With Less Bystander Activation Compared to 2D Cultures

**FIGURE 5:**
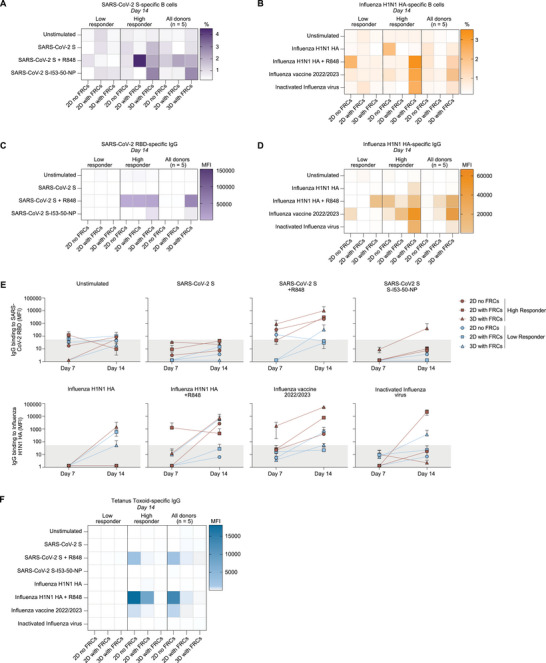
Characterization of influenza and SARS‐CoV‐2‐specific B cell responses, including analysis of antibody production, and bystander effects. Characterization of both Influenza H1N1 hemagglutinin (HA)‐ and SARS‐CoV‐2 spike‐specific B cell responses after culture. Percentages of protein‐specific cells are analyzed using flow cytometry. Protein‐specific antibody responses and bystander activation were quantified using a Luminex assay. (A) The percentage (%) of SARS‐CoV‐2 WT spike‐specific B cells out of total living CD19^+^ B cells on day 14 for a low‐responding tonsil donor (left, tonsil donor 8), a high‐responding tonsil donor (middle, tonsil donor 6), and all donors combined (right, *n* = 5 donors). The percentage of spike‐specific B cells was quantified in an unstimulated culture, or cultures stimulated with either SARS‐CoV‐2 WT spike (with and without R848) or SARS‐CoV‐2 WT‐I53‐50 NPs. All cultures were performed in 2D without FRCs, 2D with autologous FRCs, and 3D with autologous FRCs. (B) The percentage (%) of influenza H1N1 HA‐specific B cells out of total living CD19^+^ B cells on day 14 for a low‐responding tonsil donor (left, tonsil donor 8), a high‐responding tonsil donor (middle, tonsil donor 6), and all donors combined (right, *n* = 5 donors). The percentage of influenza HA‐specific B cells was quantified in an unstimulated culture, or cultures stimulated with either influenza H1N1 HA (with and without R848), influenza vaccine 2022/2023 (Influvac Tetra 2022/2023), or whole inactivated recombinant A/Netherlands/602/2009 influenza virus. All cultures were performed either in 2D without FRCs, 2D with autologous FRCs, and 3D with autologous FRCs. (C) SARS‐CoV‐2 WT receptor‐binding domain (RBD)‐specific IgG production (mean fluorescence intensity (MFI) minus blank) in the supernatants of the cultures matching the cellular data shown and described in (A). (D) Influenza H1N1 HA‐specific IgG production (mean fluorescence intensity (MFI) minus blank) in the supernatants of the cultures matching the cellular data shown and described in (B). (E) SARS‐CoV‐2 WT RBD‐specific IgG production (MFI minus blank, top row) and influenza H1N1 HA‐specific IgG production (MFI minus blank, bottom row) on day 7 vs. day 14, either unstimulated or for the varying tested antigen conditions, comparing 2D without FRCs, 2D with autologous FRCs and 3D with autologous FRCs for both the low‐ and high‐responding tonsil donors. (F) Tetanus toxoid‐specific IgG production (mean fluorescence intensity (MFI minus blank)) in the supernatants of the cultures matching the data shown and described in (A–D). (A–D; F) Data showing the mean of technical duplicates (*n* = 2), (E) data showing the mean ± SD of technical duplicates (*n* = 2).

More detailed analyses were performed on individual antigen‐specific B cell responses of each donor, comparing the three tested culture models. Overall, low percentages of antigen‐specific B cells were detected in the 2D cultures without FRCs, aligning with the observed overall low survival and activation of B cells (Figure 5A,B; Figure S5A,B). Despite the higher number of GC/ASC B cells in conditions adjuvanted with R848, there was no preferable increase in the frequency of S‐ or HA‐specific B cells, nor in S‐ and HA‐specific IgGs in the supernatants of these 2D cultures without FRCs (Figure 5A–D; Figure S5A,B,E,F). In contrast, 2D FRC‐supported cultures showed minor specific responses, mainly in the R848 adjuvanted antigen conditions. In the 3D FRC‐supported cultures, a general trend was observed where the percentage of specific B cells was higher across a broader range of tested antigen formats. At the individual level, immune responses varied substantially across donors. To capture this heterogeneity, donors were stratified into low, medium, and high responders based on antigen‐specific B‐cell frequencies and antibody levels across the 12 analysed conditions. Low responders exhibited ≤1 condition with >1.5% antigen‐specific B cells and ≤1 condition with antibody levels above the 1000 MFI threshold (one log above the detection limit). High responders showed ≥4 conditions exceeding 1.5% antigen‐specific B cells and ≥4 conditions with antibody levels above 1000 MFI, whereas medium responders fell between these two categories. In a high‐responsive donor (donor 6; Figure 5A–D), clear S‐specific responses were observed after stimulation with antigen only, as well as R848 adjuvanted antigen, S‐NPs, and influenza virus and vaccines (Figure 5A,B), with corresponding increases in S‐specific IgM (Figure S5D) and IgG production (Figure 5C). Similarly, HA‐specific responses were most pronounced following stimulation with influenza HA with R848, the 2022/2023 influenza vaccine, and inactivated influenza virus, showing superior B cell activation across more diverse antigens than in 2D cultures, both at the cellular and antibody levels (Figure 5B–D). Furthermore, there was an increase in secreted specific antibodies from day 7 to day 14, with the most pronounced responses in the 3D FRC‐supported cultures, also including notable increases among lower responding donors (Figure 5E).

Alongside quantifying increases in antigen‐specific B cells and antibodies, bystander activation was evaluated to assess non‐targeted immune responses. This included measuring S‐specific antibody production in HA‐stimulated conditions and HA‐specific antibody production in S‐stimulated conditions. Furthermore, antibodies specific to an unrelated antigen, tetanus toxoid, were quantified. An increase in tetanus toxoid‐specific IgGs was primarily observed in the R848‐adjuvanted and influenza vaccine 22/23 conditions, which generally are the conditions inducing the highest response. Interestingly, this tetanus toxoid‐specific bystander activation was mainly seen in the 2D cultures with and without FRCs, but not in the 3D FRC‐supported cultures (Figure 5F). Bystander activation leading to S‐specific antibodies in HA‐stimulated cultures, and HA‐specific antibodies in S‐stimulated cultures, were mainly observed in cultures adjuvanted with R848, regardless of the culture set‐up used. Interestingly, influenza vaccine‐stimulated cultures that also contain a broader range of immune‐activating substances did not induce these bystander activations as observed in the R848 cultures (Figure S5G–J).

### 2.5 3D FRC‐Supported Cultures Enhance Antigen‐Induced ASC Differentiation and Germinal Center‐Associated Chemokine Receptor Expression

The antigen‐induced CD38^+^ B cell responses were further analyzed across the various culture conditions, separating CD38^+^CD20^+^ GC‐like B cells from CD38^++^CD20^−^ ASCs (Figure S6A,B). Additional intracellular staining on the lymphocytic fraction at baseline confirmed the CD38^+^CD20^+^ gated GC‐like B cells as a BCL‐6 positive B cell population (Figure S6C).

**FIGURE 6:**
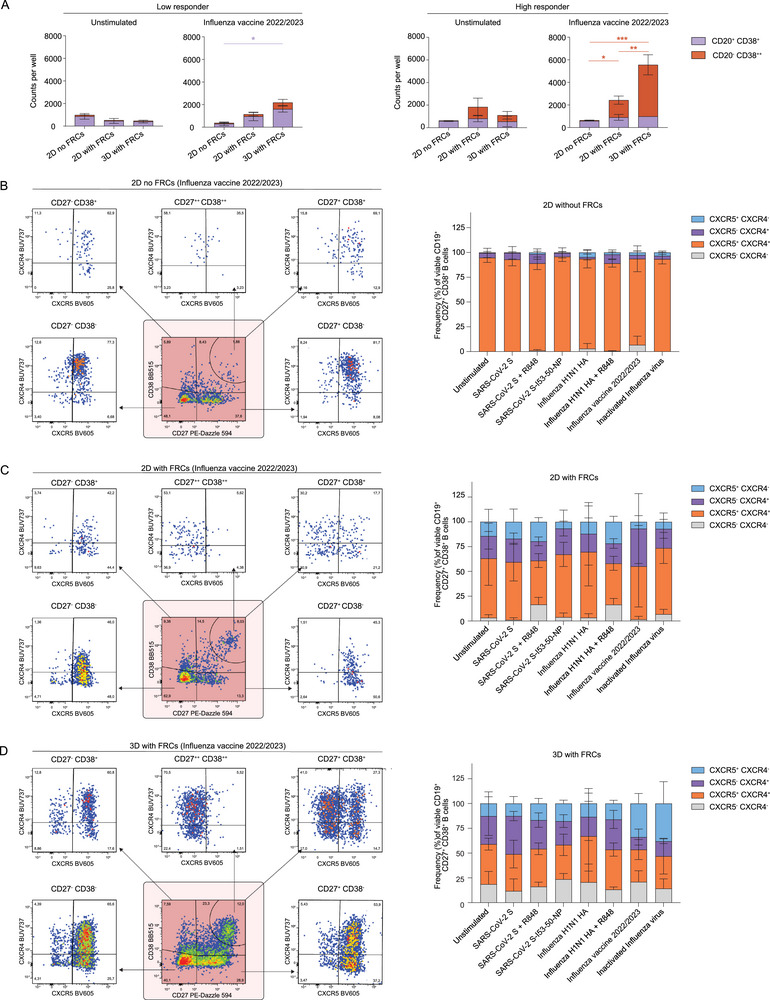
FRCs and 3D culture modulate B cell differentiation and surface chemokine expression in response to viral antigens. B cell differentiation and surface chemokine expression of B cells cultured in 2D without FRCs, 2D with autologous FRCs, or 3D with autologous FRCs, either left unstimulated or stimulated with SARS‐CoV‐2 WT spike (with and without R848), SARS‐CoV‐2 WT‐I53‐50 NP, influenza H1N1 HA (with and without R848), influenza vaccine 2022/2023 (Influvac Tetra 2022/2023) or whole inactivated recombinant A/Netherlands/602/2009 influenza virus. (A) Total counts of viable CD19^+^CD20^+^CD38^+^ B cells (GC‐like B cells) and total viable CD19^+^CD20^−^CD38^++^ B cells (ASCs) in an individual low responder (left; tonsil donor 8) and an individual high responder (right; tonsil donor 6) donor, either unstimulated or stimulated with influenza vaccine 2022/2023 (B–D) left: CXCR4 and CXCR5 chemokine expression of total living CD19^+^ B cells, subgated on either naive B cells (CD27^−^CD38^−^IgD^+^), memory B cells (CD27^+^CD38^−^), pre‐GC (CD27^−^CD38^+^), GC‐like B cells (CD27^+^CD38^+^) or antibody‐secreting cells (CD27^++^CD38^++^), of the high‐responding tonsil donor, cultured in 2D without FRCs (B), 2D with autologous FRCs (C), or 3D with autologous FRCs (D), and stimulated with influenza vaccine 2022/2023. Right: overview of the average CXCR4 and CXCR5 chemokine expression of antibody‐secreting cells (CD27^+^CD38^+^), cultured in 2D without FRCs (B), 2D with autologous FRCs (C), or 3D with autologous FRCs (D), either unstimulated or stimulated with the varying tested antigen conditions. (A) Data showing the mean ± SD of technical duplicates (*n* = 2), (B–D) data in right panels showing the mean ± SD (n = 5 tonsil donors). ^*^ = *p* < 0.05, ^**^ = *p* < 0.01, ^***^ = *p *< 0.001, ^****^ = *p* < 0.0001, statistical significance is displayed in the color of the corresponding analyzed experimental group.

In 2D cultures without FRCs, GC‐like B cells were detectable and increased significantly after R848 stimulation (*p* < 0.0001), but ASCs did not develop under any condition (Figure S6B). Addition of FRCs enabled ASC formation in 2D cultures, although this was largely confined to high‐responder donors and most evident with R848 stimulation (Figure S6B). In contrast, 3D FRC‐supported cultures consistently supported differentiation into both GC and ASC compartments across antigen formats, including S protein, S‐NPs, and HA (Figure S6B). Influenza vaccine stimulation reflected the broader donor‐dependent pattern observed across antigens (Figure S6B). In high responders, ASC counts were significantly higher in 3D cultures with FRCs compared to both 2D conditions (*p* = 0.0002 vs. 2D without FRCs; *p* = 0.0014 vs. 2D with FRCs), and 2D with FRCs also exceeded 2D without FRCs (*p* = 0.0424; Figure 6A). In low responders, 3D cultures had significantly higher GC counts than 2D cultures without FRCs (*p* = 0.005; Figure 6A). Together, these results highlight the capacity of the 3D stromal microenvironment to drive robust B‐cell differentiation into GC and ASC populations, with donor responsiveness shaping the balance between the two compartments.

Analysis of the GC‐associated chemokine receptors CXCR4 and CXCR5 on B cells revealed striking differences between the different culture set‐ups. In the 2D cultures without FRCs, B cells mainly co‐express both CXCR4 and CXCR5, with no differences for the different B cell populations identified (Figure 6B; Figure S6D). Both 2D and 3D FRC‐supported cultures contained B cell populations expressing either CXCR5 or CXCR4 alone, indicating a difference in compartmentalization in the cultures (Figure S6D). This was also evident in the different B cell subsets across stimuli, with significantly more CXCR5 single‐positive B cells among naive (CD27^−^CD38^−^) and memory B cells (CD27^+^CD38^−^), while CXCR4 single‐positive B cells were significantly enriched in the CD27^+^CD38^+^ compartment (2D + FRC vs. 2D, *p* < 0.0001; 3D + FRC vs. 2D, *p* < 0.0001; Figure 6C,D). Notably, the CD27^++^CD38^++^ population predominantly consisted of CXCR4 single‐positive B cells. This is further reflected by CD38^++^CD20^−^ ASCs, which were primarily CXCR4 single‐positive, while CD38^+^CD20^+^ GC‐like B cells contained more CXCR5 and CXCR4 double‐positive and CXCR5 single‐positive cells, indicative of DZ and LZ GC‐like B cells, respectively (Figure S6D). While 2D FRC‐supported cultures revealed no differences in chemokine receptor response comparing the varying antigen formats, the 3D FRC‐supported cultures resulted in more CXCR5 single‐positive CD27^+^CD38^+^ B cells when stimulated with both influenza vaccines (Figure 6D). This increased antigen‐induced CXCR5 upregulation and CXCR4 downregulation is mainly found on CD38^++^CD20^−^ ASCs in the 3D model, and less for CD38^+^CD20^+^ GC‐like B cells (Figure S6D).

### 2.6 Allogeneic 3D Cultures With FRCs Support Antigen‐Specific Responses Without Alloreactive Activation

**FIGURE 7:**
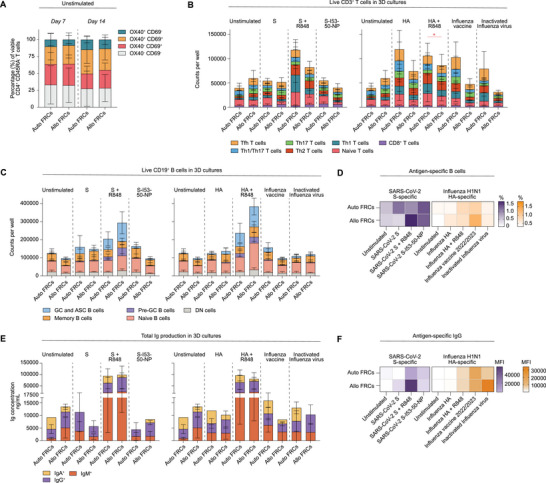
Impact of autologous vs. allogeneic FRCs on tonsil cell responses in 3D co‐cultures. (A) The percentage (%) of viable CD4^+^CD45RA^−^ T cells expressing OX40 and CD69 on day 7 (left) and day 14 (right). Co‐cultures were either 2D or 3D with autologous or allogeneic FRCs and were not stimulated with any antigen. (B) Total counts of CD8^+^ T cells, naive CD4^+^ T cells (CD45RA^+^), Th1 T cells (CD4^+^CD45RA^−^CXCR5^−^CXCR3^+^CCR6^−^), Th2 T cells (CD4^+^CD45RA^−^CXCR5^−^CXCR3^−^CCR6^−^), Th17 T cells (CD4^+^CD45RA^−^CXCR5^−^CXCR3^−^CCR6^+^), Th1/17 T cells (CD4^+^CD45RA^−^CXCR5^−^CXCR3^+^CCR6^+^) and Tfh T cells (CD4^+^CD45RA^−^CXCR5^+^) comparing both 2D and 3D autologous and allogeneic co‐cultures, cultured unstimulated, or for the varying tested antigen conditions at day 14. (C)Total counts of double‐negative cells (DN; CD27^−^CD38^−^IgD^−^), naive B cells (CD27^−^CD38^−^IgD^+^), memory B cells (CD27^+^CD38^−^), pre‐GC (CD27^−^CD38^+^), and GC and antibody‐secreting cells (GC and ASC; CD27^+^CD38^+^), comparing both 2D and 3D autologous and allogeneic co‐cultures, cultured unstimulated, or for the varying tested antigen conditions at day 14. (D)The percentage (%) of SARS‐CoV‐2 WT spike‐specific B cells (left) and influenza H1N1 HA‐specific B cells (right) out of total living CD19+ B cells on day 14. Co‐cultures were either 2D or 3D with autologous or allogeneic FRCs and were either unstimulated or stimulated with the indicated antigen conditions. (E) Total immunoglobulin (Ig) production (IgM, IgG, and IgA), quantified in the supernatant of either 2D or 3D autologous and allogeneic co‐cultures, cultured unstimulated, or for the varying tested antigen conditions. Ig concentration is in ng/mL and quantified at day 14. (F) SARS‐CoV‐2 WT RBD‐specific IgG production (left) and influenza H1N1 HA‐specific IgG production (right) measured by Luminex assay in supernatants collected on day 14. Co‐cultures were either 2D or 3D with autologous or allogeneic FRCs and were either unstimulated or stimulated with the indicated antigen conditions. MFI values are shown after subtracting the background signal (blank). Data showing the mean ± SD (*n* = 5 tonsil donors).

After establishing the beneficial role of FRCs in co‐cultures and acknowledging the time‐intensive expansion required for autologous FRCs, the use of allogeneic FRCs was explored as an alternative, and their potential was evaluated in side‐by‐side 3D co‐cultures. To exclude potential alloreactive responses induced by allogeneic stromal cells, we first assessed T cell activation markers. Expression of OX40 and CD69 on CD4^+^CD45RA^−^ T cells did not differ between autologous and allogeneic FRC co‐cultures at day 7 or 14, and CD8^+^ T cell frequencies remained unchanged, indicating no detectable alloreactive expansion (Figure 7A,B; Figure S7A,B). Across stimulations, autologous and allogeneic FRCs also supported comparable T cell viability and differentiation, including Tfh cell (CD4^+^CD45RA^−^CXCR5^+^) frequencies (Figure 7B; Figure S7B).

For B cells, viability and differentiation were also similar in autologous and allogeneic FRC co‐cultures across unstimulated and antigen‐only conditions (Figure 7C; Figure S7C). In allogeneic cultures, stimulation with HA and R848 increased naïve B cell viability (*p* = 0.027), while other B cell compartments remained unaffected (Figure 7C,D; Figure S7E,F). Antibody production was comparable between autologous and allogeneic FRC co‐cultures (Figure 7E,F; Figure S7E,F). Overall, no reproducible differences were detected in B cell responses between autologous and allogeneic FRC conditions.

Taken together, these findings show that allogeneic FRCs do not induce detectable alloreactivity and have minimal effects on B and T cell responses, supporting their use as a practical alternative to autologous FRCs in 3D co‐culture systems.

## 3 Discussion

In this study, we developed a 3D FRC‐supported in vitro model that better mimics human GC‐like responses compared to conventional 2D cultures. Our findings demonstrate that the inclusion of FRCs, particularly in a 3D matrix, enhances the survival, differentiation, and activation of B and T cells compared to conventional 2D cultures. By providing structural support within a biomimetic 3D environment, FRCs facilitated the formation of follicle‐like structures and promoted GC‐like B cell responses, as inferred by differential chemokine receptor expression, and increased frequencies of antigen‐specific B cells and differentiation to ASCs. This enhanced model not only supports antigen‐specific antibody responses but also reduces non‐specific, bystander activation, suggesting that 3D FRC‐supported cultures may serve as a valuable tool for studying human immune responses and evaluating vaccine candidates.

A 3D environment is fundamental for mimicking GC structure and function, as it better supports cell‐cell interactions compared to 2D cultures. Several studies highlighted the benefits of 3D over 2D cultures, demonstrating increased B cell survival GC‐like B cell formation, and class‐switching [18, 25, 26, 27, 28]. This study confirms that 3D cultures improve B and T cell survival [29] and differentiation compared to 2D (FRC‐supported), reinforcing the importance of a structured microenvironment for the development of adaptive immune responses [30].

However, the 3D structure alone was insufficient to mimic GC organization. Without FRCs, 3D hydrogels were unstable and disintegrated before the culture period ended. Although the RGD gel component enables integrin engagement, lymphocytes are weakly adherent, do not form mature focal adhesions, and generate minimal contractile forces, resulting in poor mechanical coupling to the network [31]. In the absence of cell‐mediated traction, the PEG matrix behaves as a freely swollen polymer and is prone to softening and fragmentation during culture [32]. In contrast, stromal cells attach, spread, and exert tension that compacts and mechanically stabilizes the gel, while depositing extracellular matrix that further reinforces the scaffold [33]. B and T cells also failed to migrate or interact effectively without the presence of FRCs, likely due to the absence of stromal‐derived guidance cues. In contrast, FRC‐supported 3D cultures facilitated the formation of well‐defined follicle‐like structures, resembling native GC organization. This structural organization is crucial, as follicle‐like aggregates promote immune cell compartmentalization, controlled antigen presentation, and efficient B‐T cell interactions, all of which are key for GC‐like immune responses [8, 34, 35].

A potential limitation of the comparison of culture set‐ups is that differences in culture outcomes may be influenced not only by the presence of FRCs but also by the plate format used for 2D cultures. The baseline 2D cultures without FRCs were performed in flat‐bottom plates following established protocols [22], while FRC‐containing cultures were adapted from previously used flat‐bottom plates [18] to U‐bottom plates to support spheroid formation. During method development, multiple formats were compared, including 2D flat‐bottom FRC‐supported cultures and 2D U‐bottom FRC‐supported cultures. We observed improved B‐cell survival in the U‐bottom FRC‐supported condition, also supported by previous optimization studies [17], although differences did not reach statistical significance. Based on these findings, the U‐bottom FRC‐supported condition was selected for further comparison with the 3D system.

While previous studies have incorporated stromal support in 3D cultures, these models primarily used mouse fibroblasts overexpressing human CD40L to mimic T cell help rather than replicating the GC stromal compartment [18, 25, 26, 27, 28]. As a result, these models lacked the natural B‐T cell interactions in an antigen‐specific context and did not support migration between GC compartments. Furthermore, many of these models rely on external cytokine supplementation to maintain cell function [25], whereas in our system, immune cells receive cytokine support from other cells in the culture, closely mimicking natural immune cell interaction in SLOs.

FRCs are not only structural components of SLOs but also active regulators of adaptive immune responses. In our 3D culture system, FRCs significantly enhanced antigen‐specific B cell frequencies and differentiation into ASCs, as well as GC‐associated chemokine receptor expression highlighting their role in B cell selection and maturation. Compared to 2D FRC‐supported cultures, 3D cultures displayed a combined response of CD38^+^CD20^+^ GC‐like B cells and CD38^++^CD20^−^ ASCs upon antigen stimulation, resulting in a significantly stronger ASC response. This aligns with previous findings that FRCs help regulate B cell positioning and influence Tfh‐B cell interactions [6, 36]. Together, these findings indicate that stromal support within a 3D microenvironment together promotes a more physiologically representative SLO‐like context, enabling the GC‐like organization, antigen‐specific immune responses, and B cell differentiation.

While still reductionist compared to the native SLO, the 3D system captures key spatial and cellular interactions that are not achievable in conventional 2D cultures. A remaining limitation of the model is the inability to comprehensively characterize antigen‐specific B cell phenotypes following culture, which would ideally provide deeper insights into their activation dynamics relative to the total CD19^+^ B cell population. This analysis is constrained by the inherently low frequency of antigen‐specific B cells. Each individual culture contained 250 000 total tonsil lymphocytes, of which 40%–70% were B cells, while antigen‐specific B cells represented on average only 0.05% of B cells. Consequently, also after antigen‐driven expansion, phenotypic characterization would yield fewer than 100 events per gate, limiting the reliability of such analyses. To avoid overinterpretation of sparse data, antigen‐specific B cell activation was therefore assessed indirectly through measurement of antigen‐specific antibody production.

Importantly, our data show that FRC‐supported 3D cultures selectively enhance antigen‐specific responses while minimizing non‐specific activation, a factor often overlooked in previous models. While other studies have reported increased antigen‐specific immune responses in their lymphoid reaggregated models, they do not distinguish whether these responses were antigen‐specific or due to general activation [21, 22, 23]. In contrast, the 2D model, representative of many conventional 2D systems, exhibits extensive bystander activation, highlighting the importance of stromal support in refining immune responses. FRCs are known to regulate immune responses by modulating dendritic cell activity, inducing T cell quiescence, and supporting regulatory T cells (Tregs), all of which contribute to immune tolerance and controlled activation [7, 9, 37]. Although Tregs were not specifically quantified, they are expected to be present in tonsil‐derived preparations across all culture conditions and may have influenced GC‐like responses, particularly Tfh‐B cell interactions [38, 39]. Combined with the compartmentalization provided by the 3D matrix, these effects may have contributed to the improved antigen specificity.

A defining feature of GCs is their compartmentalization into distinct dark and light zones, which regulate B cell selection and affinity maturation through spatially coordinated B‐T cell interactions. This organization gives rise to phenotypically distinct GC B‐cell populations: light zone (CXCR4^lo^CXCR5^+^ or CXCR4^lo^CD83^hi^CD86^hi^) and dark zone GC‐like B cells (CXCR4^hi^CXCR5^+^ or CXCR4^hi^CD83^lo^CD86^lo^) [1, 2, 3]. In this study, CXCR4 and CXCR5 expression differed between 3D FRC co‐cultures and control conditions. CD38^++^CD20^−^ ASCs displayed an increased frequency of CXCR4 single‐positive cells in the presence of FRCs, whereas CD38^+^CD20^+^ B cells contained both CXCR4^+^CXCR5^+^ and CXCR5 single‐positive populations. The presence of CXCR4 heterogeneity within the CD38^+^CD20^+^ compartment is consistent with LZ‐like (CXCR5^+^CXCR4^−^) and DZ‐like (CXCR5^+^CXCR4^+^) states and may indicate B‐cell cycling between functionally distinct niches in the FRC‐supported cultures. In addition, the lack of CXCR5 on ASCs is compatible with a phenotype ready to exit the SLO. However, CXCR4/CXCR5 expression alone is not sufficient to demonstrate bona fide GC formation, as definitive GC identity requires spatial organization, proliferation, selection dynamics, and Tfh cell dependent interactions. Therefore, these data should be interpreted as evidence for GC‐like phenotypic polarization rather than functional germinal centers. Further functional and spatial validation will be required to confirm true GC architecture and activity.

Beyond structural organization and GC‐like B cell cycling, antigen formats play a critical role in shaping immune responses. Many in vitro models rely on live‐attenuated viruses or vaccines, which are highly immunogenic and can induce broad, non‐specific immune activation (Jeger‐Madiot et al., 2024; Wagar et al., 2021). In this study, we tested multiple antigen formats with varying levels of immunogenicity, including recombinant proteins (with and without adjuvants), multivalent antigen displays, whole inactivated viruses, and commercial vaccines derived from SARS‐CoV‐2 and Influenza H1N1 (pdm09), to evaluate the model's potential as a tool for vaccine and adjuvant testing. Antigen‐specific responses varied by donor immune history and antigen format. The highest responses were observed in a donor with high baseline S‐specific B cell frequencies, emphasizing the role of pre‐existing immunity in recall responses. Among tested formulations, the Influenza vaccine 22/23 induced strong antigen‐specific activation with lower non‐specific responses, making it the most controlled immunogen. While recombinant proteins alone elicited minimal responses, recall responses in high‐responding donors suggest efficient reactivation of antigen‐experienced B cells. To support de novo responses, longer culture periods and potentially higher initial cell numbers may be necessary, reflecting the low frequency of antigen‐specific naïve B cells within the total viable B cell pool. Optimizing these parameters could extend the utility of the model to the study of primary as well as recall immune responses. Future studies should further explore the kinetics and conditions that support de novo B cell activation and differentiation.

Using this model, we also observed that stimulation with R848 (a TLR7/8 agonist) triggered strong but largely antigen‐independent responses, consistent with TLR7‐driven polyclonal B cell activation that bypasses antigen recognition [40]. While this type of innate immune activation occurs in vivo upon adjuvant administration [41], it complicates the interpretation of antigen‐specific readouts in vitro, where the specific signal becomes difficult to distinguish from background polyclonal activation, making TLR7/8 agonists suboptimal when antigen specificity is a primary readout.

One parallel advancement recently described in the field of 3D SLO modeling is the use of perfused bioreactors or chip models, culturing human tonsil tissue explant fragments, or multicellular hydrogel encapsulated tissue mimics under perfused conditions [19, 20, 21, 42]. Clear benefits of such systems have been described compared to static conditions, such as higher cell viability, increased metabolic activity, and more robust and amplified antigen‐specific immune responses. Additionally, our findings demonstrate that autologous and allogeneic FRCs perform comparably in supporting immune responses, reinforcing the scalability of this model for broader applications. The combination of a perfused culture platform, with the optimized biomimetic 3D tonsil environment described in this study would be the next step, to further assess the effect of perfusion to optimized 3D static conditions.

This study demonstrates that FRC‐supported 3D cultures provide a more physiologically relevant model for studying human GC‐like responses, surpassing conventional 2D systems. By integrating stromal support within a structured 3D microenvironment, this model enhances B and T cell survival, antigen‐specific responses, and ASC differentiation, while also supporting chemokine receptor shuttling and the formation of follicle‐like structures, both essential for GC organization and function. These findings establish FRC‐supported 3D cultures as a valuable tool for vaccine and adjuvant evaluation, as well as fundamental GC biology research.

## 4 Materials and Methods

### 4.1 Tissue Collection

Human tonsils were collected with approval by the Medical Ethical Committee of the Amsterdam UMC, Amsterdam, in accordance with the Declaration of Helsinki. Tonsils were obtained from tonsillectomies from infants to adults performed at the Onze Lieve Vrouwe Gasthuis hospital (Amsterdam, The Netherlands) as surgically discarded tissue, without accompanying information on donor age, sex, or clinical history. Therefore, we do not know if the tonsils were healthy or inflamed. The collection took place between March 2021 and May 2024.

### 4.2 Tonsil Cell Isolation

Tonsils were processed to obtain cell suspensions as published, mincing tonsils in small pieces, after which these small tissue fragments were pushed through a metal sieve with a glass plunger, collecting lymphocytes and small tissue fragments in PBS [43]. Lymphocytes were collected from this solution by density gradient centrifugation using Lymphoprep (Axis‐Shield) and cryopreserved before use in experiments. The remaining small tissue fragments were collected and plated in Dulbecco's Modified Eagle Medium high glucose (DMEM high glucose, Gibco) with 10% (v/v) fetal calf serum (FCS; Bodinco), 100 U/mL Penicillin (Life Technologies), and 100 µg/mL Streptomycin (Life Technologies). Upon outgrowth of stromal, adherent cells, a round of trypsinization was performed, removing residual tissue fragments using a 100 µm cell strainer (Corning). The resulting fibroblastic reticular cells (FRCs) were cultured for another passage until reaching confluency and cryopreserved before use in experiments.

### 4.3 Protein Production and Purification

The tetanus toxoid protein was acquired from Creative Biolabs. All protein constructs, including soluble SARS‐CoV‐2 WT spike (pre‐fusion stabilized with a T4 trimerization domain [44]), SARS‐CoV‐2 WT RBD [45], Influenza H1N1 HA (H1N1pdm2009, A/Netherlands/602/2009, GenBank: CY039527, [46]), SARS‐CoV‐2‐S‐I5350A.1NT1 plasmid [47], with or without avi‐tag, were designed as previously described. The protein production and purification of all constructs were performed as previously described [44]. See Supplementary Materials and Methods for more details.

### 4.4 Vaccines

Influvac Tetra 2022/2023 (Abbott Biologicals BV, the Netherlands) containing inactivated surface antigens (hemagglutinin (HA) and neuraminidase (NA)) of the following virus strains: A/Victoria/2570/2019 IVR‐215, A/Darwin/9/2021 SAN‐010 B/Austria/1359417/2021, BVR‐26 and B/Phuket/3073/2013 wild type (WT), containing 30 µg/mL HA of each strain. See Supplementary Materials and Methods for the production of whole inactivated recombinant A/Netherlands/602/2009 influenza virus.

### 4.5 2D and 3D Tonsil Co‐Cultures

FRCs were thawed and expanded before the start of the experiments. Tonsil lymphocytes were thawed on the day of 2D and 3D (co‐)culture plating. All conditions were performed using 250 000 tonsil lymphocytes per well. Cultures with FRCs contained 50 000 FRCs per well. For the autologous co‐cultures, donors matched with their own FRCs. For the allogeneic co‐cultures, FRCs were cross‐over paired across donors within experiments. 2D cultures without FRCs were performed in 96 Well Sphera Low‐Attachment Surface plates (ThermoFisher). 2D cultures with FRCs were performed in non‐tissue culture‐treated 96‐well U bottom plates (Greiner), in which plated FRCs formed spheroids. 3D cultures were performed in non‐tissue culture‐treated 48‐well plates (ThermoFisher). All cultures were performed using B cell medium as a basis: Iscove's Modified Dulbecco's Medium (IMDM, Gibco), 10%(v/v) fetal calf serum (FCS, Bodinco), 100 U/mL penicillin (Life Technologies), 100 µg/mL streptomycin (Life Technologies), 2 mM L‐glutamine (Life Technologies), 50 µM β‐ mercaptoethanol (Sigma–Aldrich), and 20 µg/mL Transferrin depleted for IgG (Sigma–Aldrich). Conditions with antigen stimulation contained either SARS‐CoV‐2 WT spike protein (1 µg/mL, with and without 1 µg/mL Resiquimod (R848, MedChemExpress), SARS‐CoV‐2 WT‐I53‐50 nanoparticles (1.219 µg/mL), influenza H1N1 HA protein (1 µg/mL, with and without 1 µg/mL R848), influenza vaccine 2022/2023 (Influvac Tetra 2022/2023, 1 µg/mL of total HA) or whole inactivated recombinant A/Netherlands/602/2009 influenza virus (0,01 µg/mL of total HA). 200 µL culture medium was added per well in the 96‐well plates, 400 µL culture medium was added per well in the 48‐well plates. The medium was not refreshed during the experiment. On day 7, 100 µL of fresh B cell medium was added per well. 3D cultures were performed in 15 µL PEG‐4MAL‐RGD functionalized hydrogels. All cultures were analyzed on day 0 (input lymphoid cells), 7 days after culture, and 14 days after culture. Supernatant was stored for the respective cellular fractions analysed on each time point. Results on (antigen specific) antibody concentrations in the supernatant were correct for total culture medium volume per well.

### 4.6 PEG‐4MAL Hydrogel

A cell encapsulating hydrogel was prepared as described [18, 48], using a four‐armed polyethylene glycol macromer with maleimide groups at each terminus (PEG‐4MAL, MW 20 kDa, Laysan Bio), to which cysteine‐containing adhesive peptide RGD (GRGDSPC, AAPPTec, custom synthesis, purity: >95%, trifluoroacetic acid (TFA) removal) was conjugated. The gel was crosslinked using protease degradable cross‐linking peptide GPQ‐W (GCRDGPQGIWGQDRCG, AAPPTec, custom synthesis, purity: >95%, (TFA) removal). In short: an aliquot of the PEG‐4MAL macromer, adhesive RGD peptide, and cross‐linker GPQ‐W were allowed to reach room temperature. The needed amount of GPQ‐W and RGD was weighed out and dissolved using 20 mM HEPES buffer (Sigma), after which the PH was adjusted to 7.4. Both solutions were filtered using a Costar Spin‐X centrifuge tube (Corning). The PEG‐4MAL macromer was dissolved in sterile, prefiltered 20 mM HEPES buffer. The PEG‐4MAL and RGD solution were combined in a 2:1 ratio and incubated for 15 min at 37°C. Cells were resuspended in B cell medium after which the functionalized PEG‐4MAL‐RGD precursor was added. The cross‐linking peptide solution (GPQ‐W) was added to the bottom of each well. The functionalized PEG‐4MAL‐RGD precursor mixture containing the cells was pipetted on top of the cross‐linking peptide solution and re‐suspended quickly, in a total final volume of 15 µL per well. After mixing the hydrogel, it was left cross‐linking at 37°C for 20 min, after which medium was added (illustrated in Figure 3B).

### 4.7 Cell Collection and Decrosslinking of PEG‐4MAL Hydrogel

#### 4.7.1 2D Cultures without FRCs

Cultures were resuspended and moved to a new 96‐well V‐bottom plate (ThermoFisher Scientific). After spinning down the plate, the supernatant was removed and stored at −20°C. Cells were washed two times with PBS containing 0.1% (w/v) bovine serum albumin (BSA, Sigma–Aldrich) and left in PBS containing 0.1% (w/v) BSA until further staining and analysis.

#### 4.7.2 2D Cultures with FRCs

Cultures were resuspended and moved to a new 96‐well V‐bottom plate (ThermoFisher Scientific). After spinning down the plate, the supernatant was removed and stored at −20°C. Cells were washed two times with PBS‐0.1% BSA, each time letting the plate stand for 30 s to sediment FRC spheroids. The single cell suspension above was collected and moved to a new 96‐well V‐bottom plate, removing these clumps. After washing and removal of the FRCs, cells were left in PBS‐0.1% BSA until further staining and analysis.

#### 4.7.3 3D Cultures

Supernatant was removed and stored at −20°C. Cells were washed once with PBS. Trypsin was added to the 48‐well plate and incubated for 3 min. To inactivate the trypsin, B cell medium was added in a 1:1 ratio. The trypsin diluted in B cell medium was transferred to the V‐bottom 96‐well plate, to collect free‐floating cells. Next, 200 U/mL collagenase type 1 (Gibco) diluted in B cell medium was added to each gel in the 48‐well plate. Gels were mechanically disrupted using a positive displacement pipette. The decrosslinking solution was incubated for 1 h at 37°C. All collected cells were transferred to a 96‐well V‐bottom plate (ThermoFisher Scientific). The cells in the 48‐well plate were washed two times with PBS‐0.1% BSA and every time the cells were transferred to the V‐bottom 96‐well plate. Cells of the multiple collection rounds were pooled per well. After the collection of all cells, the plate was left standing for 30 s, to let residual FRC clumps sediment. The single cell suspension above was collected and moved to a new 96‐well V‐bottom plate, removing these clumps. After this, the cells were stained for further analysis.

### 4.8 Probe Generation

Biotinylated protein antigens were individually multimerized with Alexa Fluor 647 and BV421 (both Biolegend) labeled streptavidin, as described previously [44]. In short, biotinylated proteins and fluorescently tagged streptavidin were combined in a 2:1 molar ratio of protein to fluorochrome and then incubated for 1 h at 4°C. Free streptavidin conjugates were quenched with 10 µM biotin (Genecopoiea) for a minimum of 10 min. The individually labeled proteins were then combined in equimolar amounts to achieve a final concentration of 45.5 pM.

### 4.9 Flow Cytometric Characterization

Anti‐human antibodies used for flow cytometric analysis are:

#### 4.9.1 B cell panel

Brilliant Ultraviolet 737 anti‐human CXCR4 Antibody (BD Biosciences, Clone: 12G5), Brilliant Violet 605 anti‐human CXCR5 Antibody (Biolegend, Clone: RF8B2), Alexa Fluor 700 anti‐human IgG Antibody (BD Biosciences, Clone: G18‐145), LIVE/DEAD Fixable Near IR (780) Viability Kit (Invitrogen, L34992), eFluor780 anti‐human CD3 Antibody (Invitrogen, Clone: UCHT1), eFluor780 anti‐human CD14 Antibody (Invitrogen, Clone: 61D3), eFluor780 anti‐human CD16 Antibody (Invitrogen, Clone: CB16), PE anti‐human IgA Antibody (SouthernBiotech), PE‐Dazzle 594 anti‐human CD27 Antibody (Biolegend, Clone: O323), PE‐Cy7 anti‐human IgM Antibody (Biolegend, Clone: MHM‐88), Brilliant Blue 515 anti‐human CD38 Antibody (BD Biosciences, Clone: HIT2), Brilliant Ultraviolet 395 anti‐human IgD Antibody (BD Biosciences, Clone: IA6‐2), Brilliant Ultraviolet 496 anti‐human CD19 Antibody (BD Biosciences, Clone: SJ25C1), Brilliant Ultraviolet 805 anti‐human CD20 Antibody (BD Biosciences, Clone: 2H7), and Brilliant Stain Buffer Plus (BD Horizon).

#### 4.9.2 T Cell Panel

PE‐Dazzle 594 anti‐human CD185 Antibody (CXCR5, Biolegend, Clone: J252D4), Brilliant Blue 515 anti‐human CD196 Antibody (CCR6, BD Biosciences, Clone: 11A9), Brilliant Violet 605 anti‐human CD183 Antibody (CXCR3, Biolegend, Clone: G025H7), APC anti‐human CD279 Antibody (PD‐1, Invitrogen, Clone: J105), LIVE/DEAD Fixable Near IR (780) Viability Kit (Invitrogen, L34992), PE anti‐human CD134 Antibody (OX40, BD Biosciences, Clone: ACT35), PE‐Cy7 anti‐human CD45RA Antibody (Biolegend, Clone: HI100), Brilliant Violet 421 anti‐human CD154 Antibody (CD40L, BD Biosciences, Clone: TRAP1), Brilliant Ultraviolet 395 anti‐human CD3 Antibody (BD Biosciences, Clone: HIT3a), Brilliant Ultraviolet 496 anti‐human CD4 Antibody (BD Biosciences, Clone: OKT4), Brilliant Ultraviolet 737 anti‐human CD69 Antibody (BD Biosciences, Clone: FN50), Brilliant Ultraviolet 805 anti‐human CD8 Antibody (BD Biosciences, Clone: SK1), and Brilliant Stain Buffer Plus (BD Horizon).

#### 4.9.3 FRC Panel

APC anti‐human CD45 (Beckman Coulter, Clone: J33), LIVE/DEAD Fixable Near IR (780) Viability Kit (Invitrogen, L34992), PE anti‐human CD35 (Biolegend, Clone: E11), PE‐Cy7 anti‐human PDPN (Biolegend, Clone: NC‐08), FITC anti‐human CD31 (Beckman Coulter, Clone: 5.6E), PerCP‐Efluor710 anti‐human HLA‐ABC (Invitrogen, Clone: W6/32), BV421 anti‐human HLA‐DR (BD Horizon, Clone: G46‐6), BUV395 anti‐human CD21 (BD Optibuild, Clone: B‐ly4).

#### 4.9.4 BCL‐6 Panel

PerCP‐eFluor 710 anti‐human CXCR4 Antibody (eBioscience, Clone: 12G5), Brilliant Violet 605 anti‐human CXCR5 Antibody (Biolegend, Clone: J252D4), Alexa Fluor 700 anti‐human IgG Antibody (BD Biosciences, Clone: G18‐145), LIVE/DEAD Fixable Near IR (780) Viability Kit (Invitrogen, L34992), PE anti‐human IgA Antibody (SouthernBiotech), PE‐Dazzle 594 anti‐human CD27 Antibody (Biolegend, Clone: O323), Brilliant Blue 515 anti‐human CD38 Antibody (BD Biosciences, Clone: HIT2), Brilliant Violet 421 anti‐human CD86 Antibody (BD Biosciences, Clone: 2331), Brilliant Violet 510 anti‐human IgM Antibody (Biolegend, Clone: MHM‐88), Brilliant Ultraviolet 395 anti‐human IgD Antibody (BD Biosciences, Clone: IA6‐2), Brilliant Ultraviolet 496 anti‐human CD19 Antibody (BD Biosciences, Clone: SJ25C1), Brilliant Ultraviolet 737 anti‐human CD138 Antibody (BD Horizon, Clone: ML15), Brilliant Ultraviolet 805 anti‐human CD20 Antibody (BD Biosciences, Clone: 2H7), and PE‐Cy7 anti‐human BCL6 Antibody (Biolegend, Clone: 7D1).

#### 4.9.5 Extracellular Staining Panels

Cells were resuspended in FACS buffer (PBS‐0.1% BSA) containing antibodies and, for the panels containing chemokine receptors, incubated first at 37°C with the chemokine receptor antibodies for 30 min. Next, all other antibodies were added for subsequent incubation at 4°C for 30 min. After incubation, cells were washed and fixated using 4% paraformaldehyde (PFA, Sigma). Fixated cells were washed and then re‐suspended in FACS buffer.

#### 4.9.6 Intracellular BCL‐6 Staining

After extracellular chemokine and antibody mix staining, cells were washed and fixated using Foxp3 fixation buffer (eBioscience). Foxp3 fixed samples were washed once with Foxp3 permeabilization buffer (eBioscience). Samples were stained in 25 µL staining mix containing antibodies against BCL6 and incubated overnight at 4°C. On the next day, samples were washed once with Foxp3 permeabilization buffer and resuspended in FACS buffer.

Flow cytometric data was acquired using a BD FACSymphony (Becton Dickinson).

### 4.10 Multiplex Immunoassay

The humoral response in the supernatant of tonsil cultures was measured using a customized in‐house Luminex immunoassay [49]. Briefly, HIV‐1 ConM Env v.7, tetanus toxoid, SARS‐CoV‐2 Wuhan Spike protein, SARS‐CoV‐2 Wuhan RBD, Influenza HA H1N1 PDMN2009, and a no‐protein control (75 µg each, equimolar corrected) were covalently coupled to MagPlex beads (12.5 million beads per protein) using a two‐step carbodiimide reaction. Supernatant samples were diluted 1:2 and incubated with the beads overnight, followed by detection with 1,3 ug/mL goat anti‐mouse IgM‐PE or IgG‐PE (Southern Biotech). The mean fluorescence intensity (MFI) was measured using the Magpix platform (Luminex), capturing the median signal from around 50 beads per well. Background fluorescence was accounted for by subtracting the MFI values from buffer and beads‐only controls.

### 4.11 Quantification and Statistical Analysis

Flow cytometric data were analyzed using FlowJo v10.8.1 software. The gating strategy for both the B‐cell and T‐cell panels is shown in the supplements (Figure S1). Cell counts were corrected for the original total culture volume prior to analysis. IgG, IgA, and IgM concentrations were calculated from standard curves using ELISA‐Logit‐V01Jul2018. Antigen‐specific antibody responses measured by multiplex immunoassay are expressed as MFI after background subtraction. No formal outlier exclusion criteria were applied; all donors with complete datasets were included. Data are presented as median with interquartile range or as mean ± SD, as indicated in the figure legends. Graphs were made using GraphPad Prism 9.1.1. Fluorescent z‐stack images were processed using Fiji 1.53t software to create single maximum projections. Image overviews of the 3D cultures were merged using the mosaic function of the Leica LASX software, stitching the images together using smooth and linear blending.

All statistical comparisons were performed across *n* = 5 tonsil donors using two‐sided paired Wilcoxon signed‐rank tests, as normal distribution of the data could not be assumed given the small sample size. For analyses involving multiple simultaneous comparisons, *p*‐values were corrected using the Benjamini‐Hochberg false discovery rate procedure (Q = 0.05). Statistical significance is indicated as ^*^ *p *< 0.05, ^**^ *p* < 0.01, ^***^ *p* < 0.001, ^****^ *p* < 0.0001.

## Author Contributions

Conceptualization: M.V.J.B., M.D.G., M.C., S.M.V.H., J.D.W., C.A.C.M.V.E., A.T.B., and M.J.V.G., Data Curation: M.V.J.B. and M.D.G., Formal Analysis: M.V.J.B. and M.D.G., Funding Acquisition: S.M.V.H., J.D.W., C.A.C.M.V.E., A.T.B., and M.J.V.G., Investigation: M.V.J.B., M.D.G., L.B., S.K., and T.M.B., Methodology: M.V.J.B., M.D.G., M.R., M.C., S.M.V.H., J.D.W., C.A.C.M.V.E., A.T.B., and M.J.V.G., Project Administration: M.V.J.B., M.D.G., M.C., S.M.V.H., J.D.W., C.A.C.M.V.E., A.T.B., and M.J.V.G., Resources: M.R., S.M.V.H., J.D.W., C.A.C.M.V.E., A.T.B., and M.J.V.G., Supervision: M.C., S.M.V.H., J.D.W., C.A.C.M.V.E., A.T.B., and M.J.V.G., Validation: M.V.J.B., M.D.G., A.T.B., and M.J.V.G., Visualization: M.V.J.B. and M.D.G., Writing – Original Draft: M.V.J.B. and M.D.G., Writing – Review and Editing: All Authors.

## Funding

This work was supported by the Netherlands Organization for Scientific Research (NWO) grants (OCENW.KLEIN.479 and Aspasia‐015.014.070) to M.v.G, and by Amsterdam UMC through the AMC Fellowship (2017) to M.v.G. Part of this research was supported by the Target‐to‐B consortium to M.V.J.B, a collaboration project that is financed by the PPP Allowance made available by Top Sector Life Sciences & Health to Samenwerkende Gezondheidsfondsen (SGF) in the Netherlands under project number LSHM18055‐SGF to stimulate public‐private partnerships and co‐financing by health foundations that are part of the SGF. We acknowledge the support of patient partners, private partners, and active colleagues of the Target‐to‐B consortium (see website: www.target‐to‐b.nl). The funders had no role in study design, data collection and analysis, decision to publish, or preparation of the article.

## Conflicts of Interest

The authors declare no conflicts of interest.

## Supporting information




**Supporting File**: adhm71150‐sup‐0001‐SuppMat.pdf.

## Data Availability

The data that support the findings of this study are available from the corresponding author upon reasonable request.
