# Supplementary material for: Enhanced Human Antigen‐Specific B Cell Responses Using In Vitro 3D Tonsil Cultures Containing Stromal Cells
Source: Adv Healthc Mater. 2026 Apr 16;15(22):e04886. doi: 10.1002/adhm.202504886 (PMC13279853; doi:10.1002/adhm.202504886)
Supplement: Supplementary file 1 — Supporting File: adhm71150‐sup‐0001‐SuppMat.pdf. [file ADHM-15-0-s001.pdf]

Enhanced Human Antigen-Specific B Cell Responses Using *in vitro* 3D Tonsil Cultures Containing Stromal Cells

Maaike V.J. Braham[#,1,2,4], Marlon de Gast[#,2,3] Liubov Babii[1,2], Sabine Kruijer[2,3], Theo M. Bestebroer[5], Mathilde Richard[5], Mathieu Claireaux[2,3], S. Marieke van Ham[1,2,6], Jelle de Wit[4], Cécile A.C.M. van Els[4,7], Anja ten Brinke[*,1,2], Marit J. van Gils[*,2,3]

Corresponding authors: Anja ten Brinke and Marit J. van Gils (a.tenbrinke@sanquin.nl and m.j.vangils@amsterdamumc.nl)

This PDF file includes:

Supplementary Materials and Methods
Supplementary Figures S1 – S7

Supplementary Materials and Methods

Protein production and purification

The tetanus toxoid protein was acquired from Creative Biolabs. All protein constructs, including soluble SARS-CoV-2 WT spike (pre-fusion stabilized with a T4 trimerization domain [43]), SARS-CoV-2 WT RBD [44], Influenza H1N1 HA (H1N1pdm2009, A/Netherlands/602/2009, GenBank: CY039527 [45]), SARS-CoV-2-S-I5350A.1NT1 plasmid [46], with or without avi-tag, were designed as previously described. The protein production and purification of all constructs were performed as previously described [43]. For protein production, HEK 293F cells (Invitrogen) were maintained in Freestyle medium (Life Technologies) at a density of 0.8-1.2 million cells/mL. Cells were transiently transfected with the respective expression plasmids using polyethylenimine hydrochloride (PEI) MAX (Polysciences). The transfection mixture consisted of PEI MAX at a concentration of 1 mg/mL and expression plasmids at 312.5 μg/L in a 3:1 ratio (PEI MAX:plasmid) in OptiMEM (Gibco).

This mixture was added to the cells and six days after transfection, supernatants were harvested by centrifugation at 4000 rpm for 30 minutes at 4 degrees. Supernatants were filtered using 0.22 μm Steritop filters (Merck Millipore). His-tagged proteins were purified from the clarified supernatant by affinity chromatography using Ni-NTA agarose beads (QIAGEN). Following elution, proteins were concentrated, and buffer exchanged into PBS using Vivaspin centrifugal concentrators with a 100 kDa molecular weight cutoff (MWCO) (GE Healthcare). After purification, Avi-tagged proteins were biotinylated using a BirA500 biotin-ligase reaction kit (Avidity), according to the manufacturer's instructions. To remove unbound biotin, the proteins were further purified using size-exclusion chromatography (SEC) on a Superdex 200 16/60 column (Cytiva), with PBS as the elution buffer.

For the assembly of SARS-CoV-2 WT Spike I53-50 nanoparticles (NP), the purified SARS-CoV-2-S-I5350A.1NT1 fusion proteins were buffer exchanged into Tris-buffered saline (TBS) and sterilized using a 0.22 μm spin column. NP assembly was performed as previously described [46]. Briefly, the S-I53-50A.1NT1 proteins were further purified by size-exclusion chromatography (SEC) on a Superose 6 increase 10/300 GL column (GE Healthcare) in TBS with 5% glycerol. The appropriate fractions were collected and pooled. Equimolar amounts of

I53-50B.4PT1 were mixed with the S-I53-50A.1NT1 protein and incubated overnight to allow NP assembly. Assembled NPs were then purified by SEC to remove any unassembled components, and the relevant fractions were concentrated using a 10.000 Da Vivaspin column (GE healthcare).

Protein concentrations of all proteins were determined using a Nanodrop 2000 Spectrophotometer using the protein's peptidic molecular weight.

Whole inactivated recombinant A/Netherlands/602/2009 influenza virus

293T cells (ATCC) were cultured in Dulbecco modified Eagle's medium (DMEM) (Capricorn Scientific) supplemented with 10% fetal calf serum (FCS) (Sigma-Aldrich),1x non-essential amino acids (Capricorn Scientific), 1 mM sodium pyruvate (Gibco), 2 mM L-glutamine (Capricorn Scientific), 100 U/mL penicillin and 100 U/mL streptomycin (Capricorn Scientific), and 0.5 mg/mL Geneticin (Gibco). Madin-Darby canine kidney (MDCK) cells (ATCC) were cultured in Eagle's minimal essential medium (EMEM, Capricorn Scientific), supplemented with 10% FCS, 1x non-essential amino acids (Capricorn Scientific), 1.5 mg/mL sodium bicarbonate (Gibco), 10 mM HEPES (Capricorn Scientific), 2 mM L-glutamine (Capricorn Scientific), 100 U/mL penicillin and 100 U/mL streptomycin (Capricorn Scientific). Cells were cultured at 37 °C, 5% CO2, and passaged twice weekly.

The A/Netherlands/602/2009 recombinant influenza virus was generated by reverse genetics using eight bidirectional plasmids as described previously (Siegers et al. 2023). It contained the hemagglutinin and neuraminidase of the A/Netherlands/602/2009 virus and the remaining six genes of A/Puerto-Rico/8/1934. Briefly, recombinant virus was produced by transfecting bidirectional plasmids into 293T cells using the calcium-phosphate transfection method. Approximately 16 hours after transfection, the cells were washed once with PBS and fresh media containing 2% FCS. Three days after transfection, supernatant dilutions from 293T cells were used to inoculate MDCK cells. Virus stock production in MDCK cells was performed using EMEM medium containing the same supplements as described above, but without FCS and with the addition of 20-35 mg/mL of N-tosyl-L-phenylalanine chloromethyl ketone (TPCK)-treated trypsin (Sigma-Aldrich), referred to as infection medium. MDCK supernatant was harvested two to three days post-inoculation and centrifuged at 2,100 g for 10 minutes to remove cellular debris. The presence of virus was confirmed by hemagglutination (HA) assays

using 1% turkey red blood cells (TRBCs, harvested from in-house turkeys) in PBS. The sequences of all plasmids, along with the hemagglutinin and neuraminidase genes of A/Netherlands/602/2009 recombinant viruses, were confirmed with Sanger sequencing using the BigDye™ Terminator v3.1 Cycle Sequencing Kit (Applied Biosystems) and the 3500xL Genetic Analyzer (Applied Biosystems).

Whole-inactivated virus vaccines were generated as follows. Eleven-day old embryonated chicken eggs were inoculated with the A/Netherlands/602/2009 recombinant influenza virus. Allantoic fluid was harvested two days post-inoculation and centrifuged for ten minutes at 2,100 g to remove cellular debris. Subsequent centrifugation steps were performed at 124,000 g (SW 32 Ti, Beckman Coulter) at 4 °C, unless indicated otherwise. The allantoic fluid was concentrated on a 60% sucrose cushion by centrifuging for 2 hours. Following this, resuspended sucrose cushions from multiple tubes were pooled and loaded on 60-50-40-30-20% sucrose gradients, which were centrifuged overnight at the lowest deceleration setting. The virus band, located on top of the 30% sucrose layer, was harvested, diluted in PBS, and subsequently pelleted by centrifugation for 2 hours to remove the sucrose. The pellet was dissolved in PBS. The dissolved pellets were transferred to dialysis chambers (Slide-A-Lyzer™ Dialysis Cassettes, 10K MWCO, Thermo Fisher Scientific) which were subsequently submerged in PBS containing 0.01% formalin for three days. The dialysis chambers were then submerged in PBS for a day, during which the PBS was refreshed twice. The resulting vaccines were aliquoted and stored at -80 °C. Vaccine inactivation was confirmed by two serial blind passages on MDCK cells. Total protein content was determined using the Pierce BCA total protein analysis kit (Thermo Fisher Scientific). The absolute HA content was estimated from non-reducing SDS-PAGE protein gels using dilutions of a A/Netherlands/602/2009 hemagglutinin recombinant protein as standard and stained with instant Blue (Expedeon).

Immunocytochemistry

3D cultures were washed with PBS and then fixated in 4% PFA for 1 hour. Samples were washed with PBS and blocked with 5% (w/v) BSA in PBS for 24 hours at 4°C on a plate shaker (100 1/min) and subsequently washed in PBS. Immunodetection was performed by incubation with purified mouse anti-human CD19 antibody (14.3 ug/mL, HIB19, BD Biosciences) and

purified rat anti-human CD4 antibody (14.3 ug/mL, A161A1, Biolegend) for the first panel and by incubation with purified mouse anti-human CD185 (CXCR5) antibody (14.3 ug/mL, J252D4, Biolegend), recombinant rabbit anti-CXCR4 antibody (77.4 ug/mL, UMB2, Abcam) and rat CD19 monoclonal antibody (14.3 ug/mL, 6OMP31, Invitrogen) for the second panel for 72h at 4°C. As a second step, goat anti-mouse IgG (minimal x-reactivity) Alexa Fluor 488 (40 ug/mL, 405319, Biolegend), goat anti-rat IgG (minimal x-reactivity) Alexa Fluor 555 (40 ug/mL, 405420, Biolegend) were added for both panels, with additionally goat anti-rabbit IgG H&L Alexa Fluor® 647 (40 ug/mL, ab150079, Abcam) for the second panel for 72 hour at 4°C, all in PBS-0.1% BSA and incubated on a plate shaker (100 1/min). All antibodies were centrifuged before use (13.000 rpm for 5 min). Between staining steps, samples were washed in 0.1% Tween 20 (v/v, Merck Millipore) in PBS for 2 hours (first wash) and for 24 hours at 4°C on a plate shaker (100 1/min, second wash). Samples were then stained for DAPI (100 ng/mL, D9542, Sigma Aldrich) and Phalloidin Alexa Fluor™ 647 (165 nM, A22287, Invitrogen™) for the first panel, and DAPI and Phalloidin-Atto 700 (0.4 nmol/mL, 79286, Sigma Aldrich) for the second panel for 24 h at 4°C. Then samples were washed twice in PBS, with a final washing step overnight at 4°C on a plate shaker (100 1/min). All samples were mounted on microscopy slides using Vectashield (Vector Laboratories, H-1700-10).

Confocal Imaging

Confocal images were taken with a Leica TCS SP8 or a Leica Stellaris 8 confocal microscope using Leica LASX acquisition software. On the SP8, four detection channels collected the fluorescent signal from the used fluorochromes: DAPI, Alexa Fluor 488, Alexa Fluor 555 and Alexa Fluor 647, which were given the pseudocolors cyan, green, yellow, and red. On the Stellaris 8, five detection channels collected the fluorescent signal from the used fluorochromes: DAPI, Alexa Fluor 488, Alexa Fluor 555, Alexa Fluor 647, and Phalloidin–Atto 700, which were given the pseudocolors blue, green, yellow, cyan and red.

ELISA

96-well Nunc Maxisorp plates (ThermoFisher Scientific) were coated with 2 ug/ml monoclonal mouse anti-human IgG (MH16-1, Sanquin), 1 ug/ml monoclonal mouse anti-human IgA (MH14-1, Sanquin) or 2 ug/ml monoclonal mouse anti-human IgM (MH15-1, Sanquin) diluted in PBS and incubated overnight at RT. The plates were washed 5 times with PBS-Tween (10%

(v/v)). The supernatants of the cultures were thawed and diluted 1:25, 1:125, 1:625 in High-Performance ELISA buffer (HPE buffer, Sanquin) and added to the plates. A standard curve of a serum pool with known antibody concentrations (Pool serum 15-123, Sanquin) was diluted in HPE and also added to the plate, as well as 2 blanks. The plates were incubated for 1 hour at RT on a shaker and washed afterwards. Then, 1 ug/ml monoclonal mouse anti-human IgG HRP (MH16-1, Sanquin), 1 ug/ml monoclonal mouse anti-human IgA HRP (MH14-1, Sanquin), or 1 ug/ml monoclonal mouse anti-human IgM (MH15-1, Sanquin), all diluted to the final concentrations in HPE, were added to the plate and incubated for 1 hour at RT on a shaker. The plates were washed and the substrate, 1-step Ultra TMB-ELISA (Thermo Fisher Scientific) diluted in MiliQ in a ratio of 1:1, was added to the plate. The reaction was stopped with 0.2 M H_2SO_4, measured at 450 nm and corrected for absorbance background at 540 nm with a Synergy 2 microplate reader (Biotek Instruments Inc.).

Figure S1

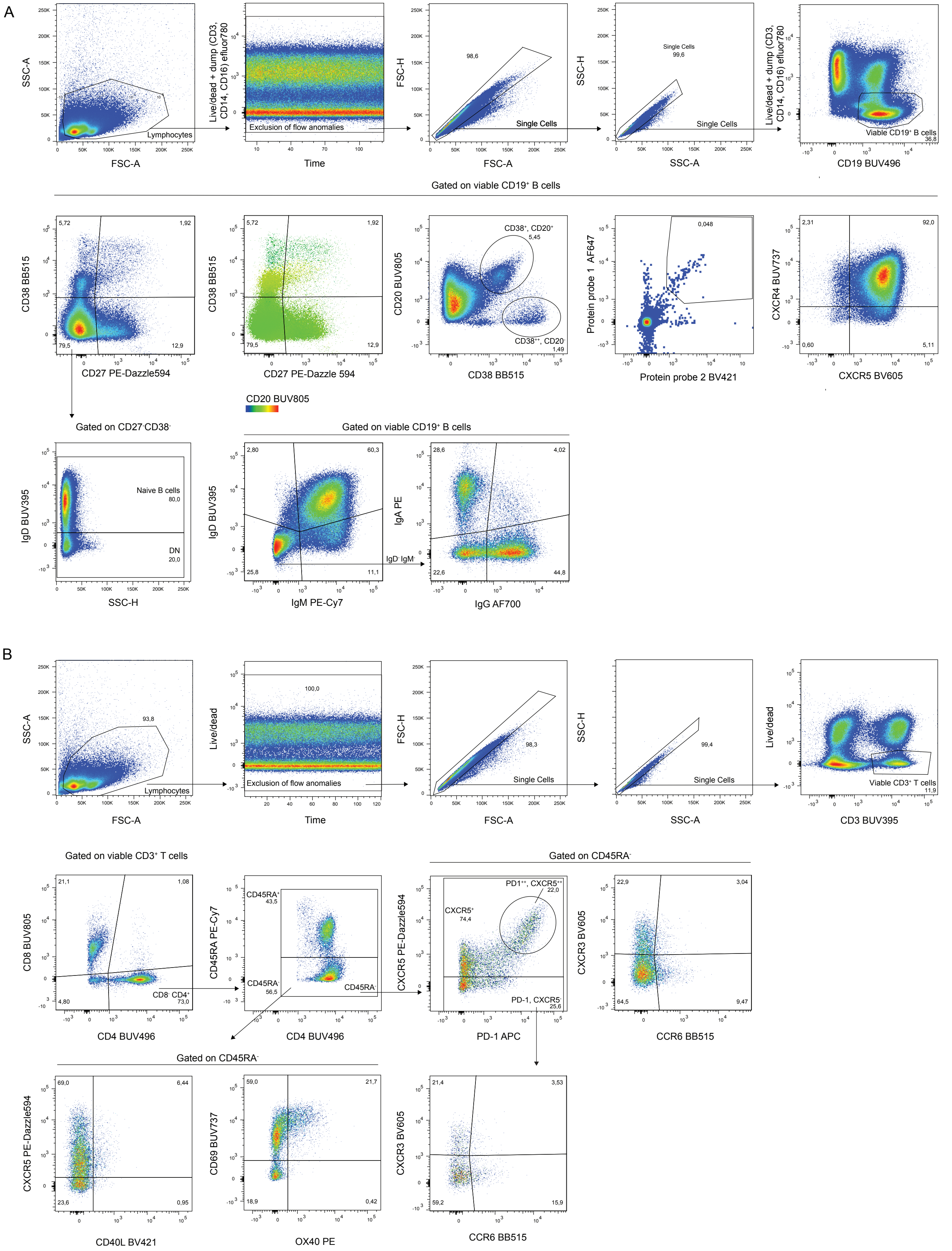

A
SSC-A
FSC-A
Lymphocytes
Live/dead + dump (CD3, CD14, CD16) efluor780
Time
Exclusion of flow anomalies
FSC-H
98,6
Single Cells
SSC-H
Single Cells
99,6
SSC-A
CD19 BUV496
Viable CD19+ B cells
36,8
Gated on viable CD19+ B cells
CD38 BB515
CD27 PE-Dazzle594
5,72
1,92
79,5
12,9
CD27 PE-Dazzle 594
CD20 BUV805
CD38+, CD20+
5,45
CD38++, CD20-
1,49
Protein probe 1 AF647
Protein probe 2 BV421
0,048
CXCR4 BUV737
CXCR5 BV605
2,31
92,0
0,60
5,11
Gated on CD27-CD38-
IgD BUV395
Naive B cells
80,0
DN
20,0
SSC-H
IgM PE-Cy7
2,80
60,3
25,8
11,1
IgD-IgM-
IgA PE
IgG AF700
28,6
4,02
22,6
44,8
B
93,8
Live/dead
100,0
98,3
99,4
CD3 BUV395
Viable CD3+ T cells
11,9
Gated on viable CD3+ T cells
CD8 BUV805
CD4 BUV496
21,1
1,08
4,80
CD8- CD4+
73,0
CD45RA PE-Cy7
CD45RA+
43,5
CD45RA-
56,5
Gated on CD45RA-
CXCR5 PE-Dazzle594
PD-1 APC
PD1++, CXCR5++
22,0
CXCR5+
74,4
PD-1, CXCR5-
25,6
CXCR3 BV605
CCR6 BB515
22,9
3,04
64,5
9,47
CD40L BV421
69,0
6,44
23,6
0,95
CD69 BUV737
OX40 PE
59,0
21,7
18,9
0,42
21,4
3,53
59,2
15,9

Figure S1. Flow cytometry gating strategy for tonsil lymphocytes. Flow cytometry staining and gating strategy, showing representative data of tonsil lymphocytes analyzed for B cells **(A)** and T cells **(B)** on day 0. All samples were gated on lymphocytes, and flow anomalies, doublets and dead cells were excluded from the analysis. The phenotypical B cell populations (memory B cells ($CD27^+CD38^-$), pre-GC ($CD27^-CD38^+$), and GC and antibody-secreting cells (GC and ASC; $CD27^+CD38^+$) were gated from the viable $CD19^+$ B cell gate. To discriminate between naive B cells ($CD27^-CD38^-IgD^+$) and DN B cells ($CD27^-CD38^-IgD^-$), an additional IgD gating was used. The phenotypical T cell populations were gated from the viable $CD45RA^-$ T cell gate.

Figure S2

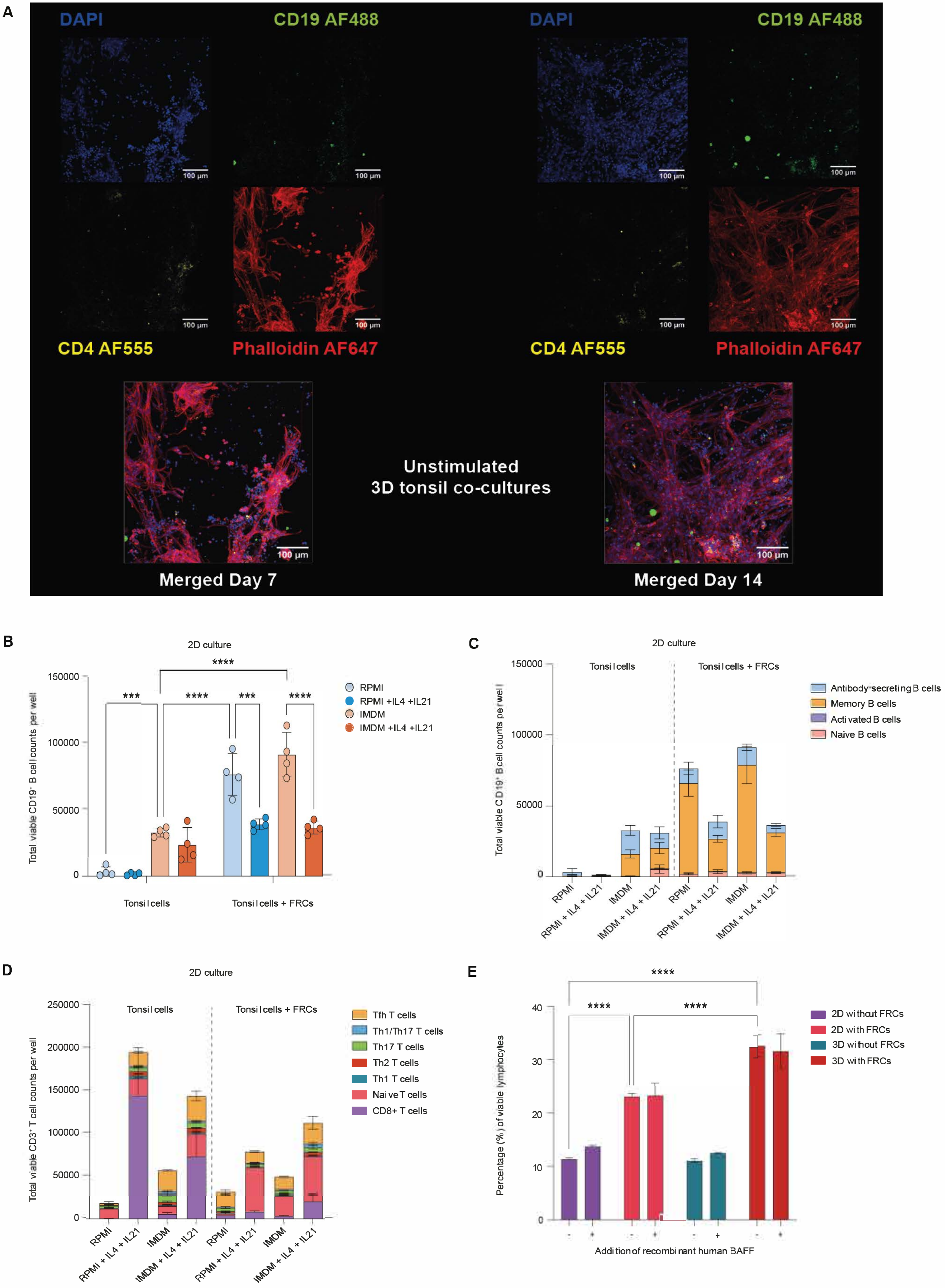
A
DAPI
CD19 AF488
CD4 AF555
Phalloidin AF647
100 µm
Unstimulated
3D tonsil co-cultures
Merged Day 7
Merged Day 14
B
2D culture
Total viable CD19⁺ B cell counts per well
150000
100000
50000
0
***
****
****
***
****
RPMI
RPMI +IL4 +IL21
IMDM
IMDM +IL4 +IL21
Tonsil cells
Tonsil cells + FRCs
C
2D culture
Tonsil cells
Tonsil cells + FRCs
Total viable CD19⁺ B cell counts per well
150000
100000
50000
0
Antibody-secreting B cells
Memory B cells
Activated B cells
Naive B cells
RPMI
RPMI + IL4 + IL21
IMDM
IMDM + IL4 + IL21
D
2D culture
Tonsil cells
Tonsil cells + FRCs
Total viable CD3⁺ T cell counts per well
250000
200000
150000
100000
50000
0
Tfh T cells
Th1/Th17 T cells
Th17 T cells
Th2 T cells
Th1 T cells
Naive T cells
CD8+ T cells
RPMI
RPMI + IL4 + IL21
IMDM
IMDM + IL4 + IL21
E
****
****
****
Percentage (%) of viable lymphocytes
40
30
20
10
0
2D without FRCs
2D with FRCs
3D without FRCs
3D with FRCs
-
+
Addition of recombinant human BAFF

Figure S2. Confocal imaging of unstimulated 3D tonsil co-cultures with FRCs and culture optimization of unstimulated tonsil cells, using varying (co-)culture conditions, basal media, and cytokine supplementation.

(A) Confocal imaging of 3D tonsil co-cultures with FRCs. Blue = DAPI, green = CD19 Alexa fluor 488, yellow = CD4 Alexa fluor 555, red = phalloidin Alexa fluor 647. Left image: unstimulated culture on day 7. Right image: unstimulated culture on day 14. Scale bars represent 100µm. **(B)** Total viable $CD19^+$ B cell counts on day 14, comparing 2D cultured tonsil cells without FRCs to 2D cultured tonsil cells with FRCs, either in RPMI versus IMDM, with or without additional supplementation of B cell cytokines IL4 and IL21 (both 50 ng/mL). **(C)** Total counts of naive B cells ($CD27^-CD38^-IgD^+$), memory B cells ($CD27^+CD38^-$), pre-GC ($CD27^-CD38^+$), and GC and antibody-secreting cells (GC and ASC; $CD27^+CD38^+$) either cultured in 2D without FRCs, or 2D with FRCs, in RPMI versus IMDM, with or without additional supplementation of B cell cytokines IL4 and IL21. **(D)** Total counts of $CD8^+$ T cells, naive $CD4^+$ T cells ($CD45RA^+$), Th1 T cells ($CD4^+CD45RA^-CXCR5^-CXCR3^+CCR6^-$), Th2 T cells ($CD4^+CD45RA^-CXCR5^-CXCR3^-CCR6^-$), Th17 T cells ($CD4^+CD45RA^-CXCR5^-CXCR3^-CCR6^+$), Th1/17 T cells ($CD4^+CD45RA^-CXCR5^-CXCR3^+CCR6^+$) and Tfh T cells ($CD4^+CD45RA^-CXCR5^+$) either cultured in 2D without FRCs, or 2D with FRCs, in RPMI versus IMDM, with or without additional supplementation of B cell cytokines IL4 and IL21. **(E)** Percentage (%) of viable lymphocytes when cultured in 2D without FRCs, 2D with FRCs, 3D without FRCs, or 3D with FRCs, comparing the effect of additional 1µg/mL recombinant human B cell-activating factor (BAFF) supplementation in IMDM medium without IL4 and IL21 supplementation.

Data showing the mean ± SD (n=4 tonsil donors).

Figure S3

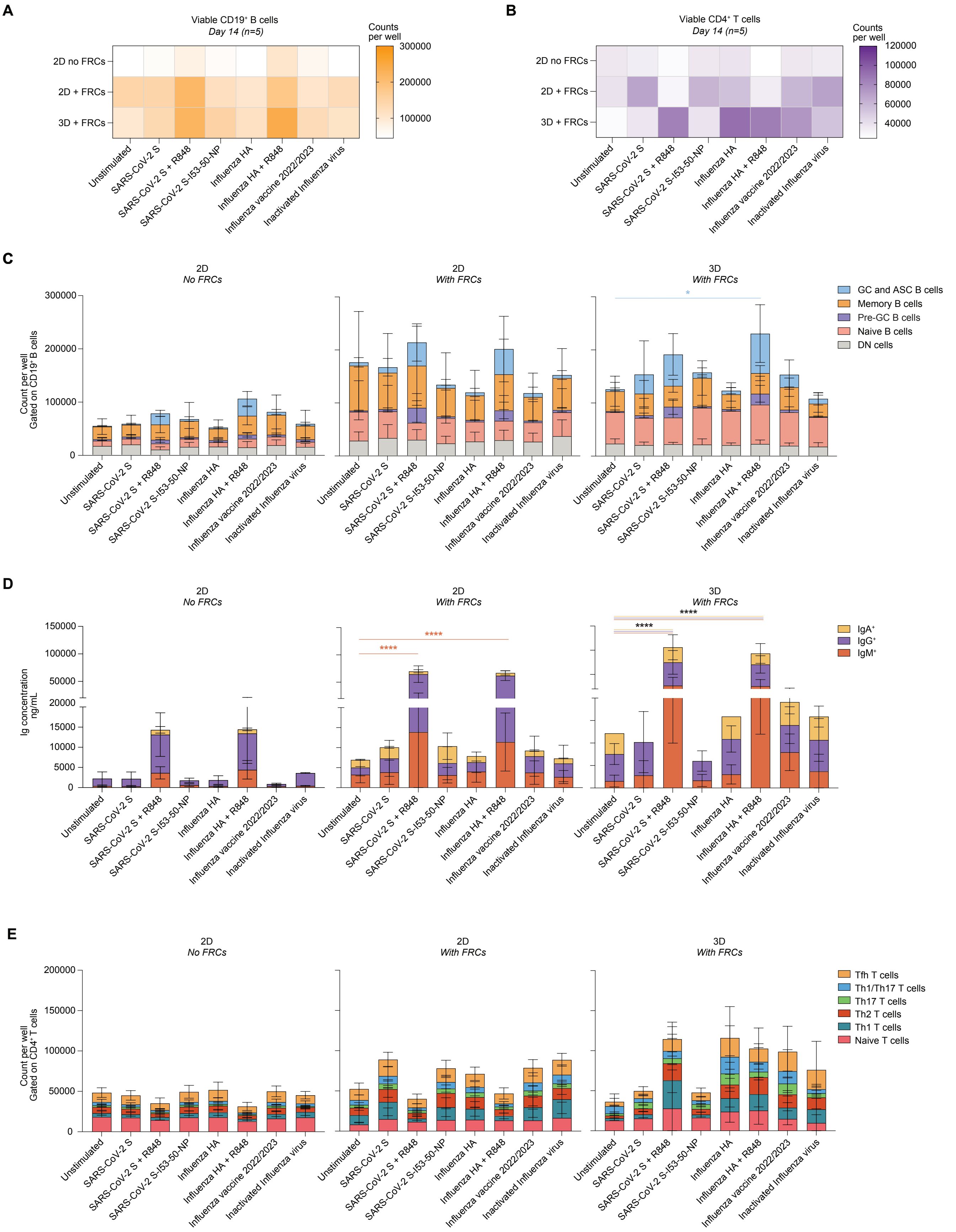
A
Viable CD19⁺ B cells
Day 14 (n=5)
Counts per well
300000
200000
100000
2D no FRCs
2D + FRCs
3D + FRCs
Unstimulated
SARS-CoV-2 S
SARS-CoV-2 S + R848
SARS-CoV-2 S-I53-50-NP
Influenza HA
Influenza HA + R848
Influenza vaccine 2022/2023
Inactivated Influenza virus
B
Viable CD4⁺ T cells
Day 14 (n=5)
Counts per well
120000
100000
80000
60000
40000
C
2D
No FRCs
2D
With FRCs
3D
With FRCs
Count per well
Gated on CD19⁺ B cells
300000
200000
100000
0
*
GC and ASC B cells
Memory B cells
Pre-GC B cells
Naive B cells
DN cells
D
Ig concentration
ng/mL
150000
100000
50000
20000
15000
10000
5000
0
****
IgA⁺
IgG⁺
IgM⁺
E
Count per well
Gated on CD4⁺ T cells
200000
150000
100000
50000
0
Tfh T cells
Th1/Th17 T cells
Th17 T cells
Th2 T cells
Th1 T cells
Naive T cells

Figure S3. Impact of FRCs and 3D culture on B and T cell survival, differentiation, and immunoglobulin production in response to viral antigens. Survival of B and T cells when cultured in 2D without FRCs, 2D with autologous FRCs, or 3D with autologous FRCs, either left unstimulated or stimulated with SARS-CoV-2 WT spike protein (with and without R848), SARS-CoV-2 WT-I53-50 nanoparticles (NP), influenza H1N1 HA protein (with and without R848), influenza vaccine 2022/2023 (Influvac Tetra 2022/2023) or whole inactivated recombinant A/Netherlands/602/2009 influenza virus. Total viable B and T cell numbers are analyzed, as well as differentiation of B and T cells and total immunoglobulin production. **(A)** Viable $CD19^+$ B cell counts on day 14 of each tested culture condition. **(B)** Viable $CD4^+$ T cell counts on day 14 of each tested culture condition. **(C)** Total counts of double-negative cells (DN; $CD27^-CD38^-IgD^-$), naive B cells ($CD27^-CD38^-IgD^+$), memory B cells ($CD27^+CD38^-$), pre-GC ($CD27^-CD38^+$), and GC and antibody-secreting cells (GC and ASC; $CD27^+CD38^+$) either cultured in 2D without FRCs (left), 2D with FRCs (middle) or 3D with FRCs (right) cultured unstimulated, or for the varying tested antigen conditions at day 14. **(D)** Total immunoglobulin (Ig) production (IgM, IgG, and IgA) in the supernatant of tonsil cultures on day 14. Cultures were in 2D without FRCs (left), 2D with FRCs (middle), or 3D with FRCs (right), either unstimulated or stimulated with the various antigen conditions. Ig concentration is in ng/mL. **(E)** Total counts of naive $CD4^+$ T cells ($CD45RA^+$), Th1 T cells ($CD4^+CD45RA^-CXCR5^-CXCR3^+CCR6^-$), Th2 T cells ($CD4^+CD45RA^-CXCR5^-CXCR3^-CCR6^-$), Th17 T cells ($CD4^+CD45RA^-CXCR5^-CXCR3^-CCR6^+$), Th1/17 T cells ($CD4^+CD45RA^-CXCR5^-CXCR3^+CCR6^+$) and Tfh T cells ($CD4^+CD45RA^-CXCR5^+$) either cultured in 2D without FRCs (left), 2D with FRCs (middle), or 3D with FRCs (right) cultured unstimulated, or for the varying tested antigen conditions at day 14.

(A and B) Data showing the mean (n=5 tonsil donors), (C-E) Data showing the mean ± SD (n=5 tonsil donors). *=p<0.05, **=p<0.01, ***=p<0.001, ****=p<0.0001, statistical significance is displayed in the color of the corresponding analyzed experimental group.

Figure S4

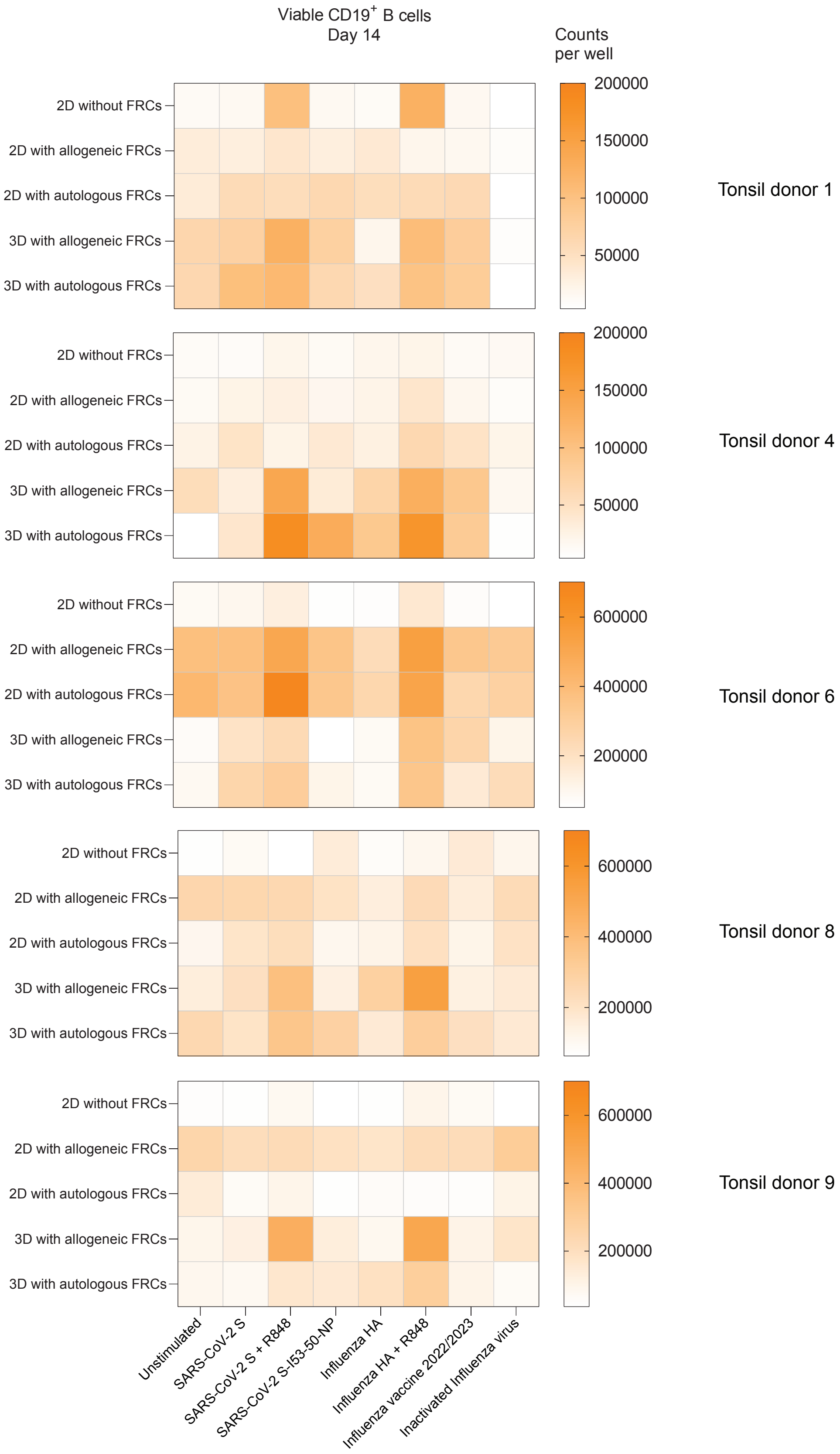
Viable CD19+ B cells
Day 14
Counts
per well
Tonsil donor 1
Tonsil donor 4
Tonsil donor 6
Tonsil donor 8
Tonsil donor 9
2D without FRCs
2D with allogeneic FRCs
2D with autologous FRCs
3D with allogeneic FRCs
3D with autologous FRCs
200000
150000
100000
50000
600000
400000
200000
Unstimulated
SARS-CoV-2 S
SARS-CoV-2 S + R848
SARS-CoV-2 S-I53-50-NP
Influenza HA
Influenza HA + R848
Influenza vaccine 2022/2023
Inactivated Influenza virus

Figure S4. Influence of FRCs and culture dimensionality on B cell survival. Survival of B cells when cultured in 2D without FRCs, 2D with autologous FRCs, 2D with allogeneic FRCs, 3D with autologous FRCs or 3D with allogeneic FRCs, either left unstimulated or stimulated with SARS-CoV-2 WT spike (with and without R848), SARS-CoV-2 WT-I53-50 nanoparticles (NP), influenza H1N1 HA (with and without R848), influenza vaccine 2022/2023 (Influvac Tetra 2022/2023) or whole inactivated recombinant A/Netherlands/602/2009 influenza virus. Total viable B cell counts are shown for each individual donor tested, in all studied culture conditions, at day 14.

Figure S5

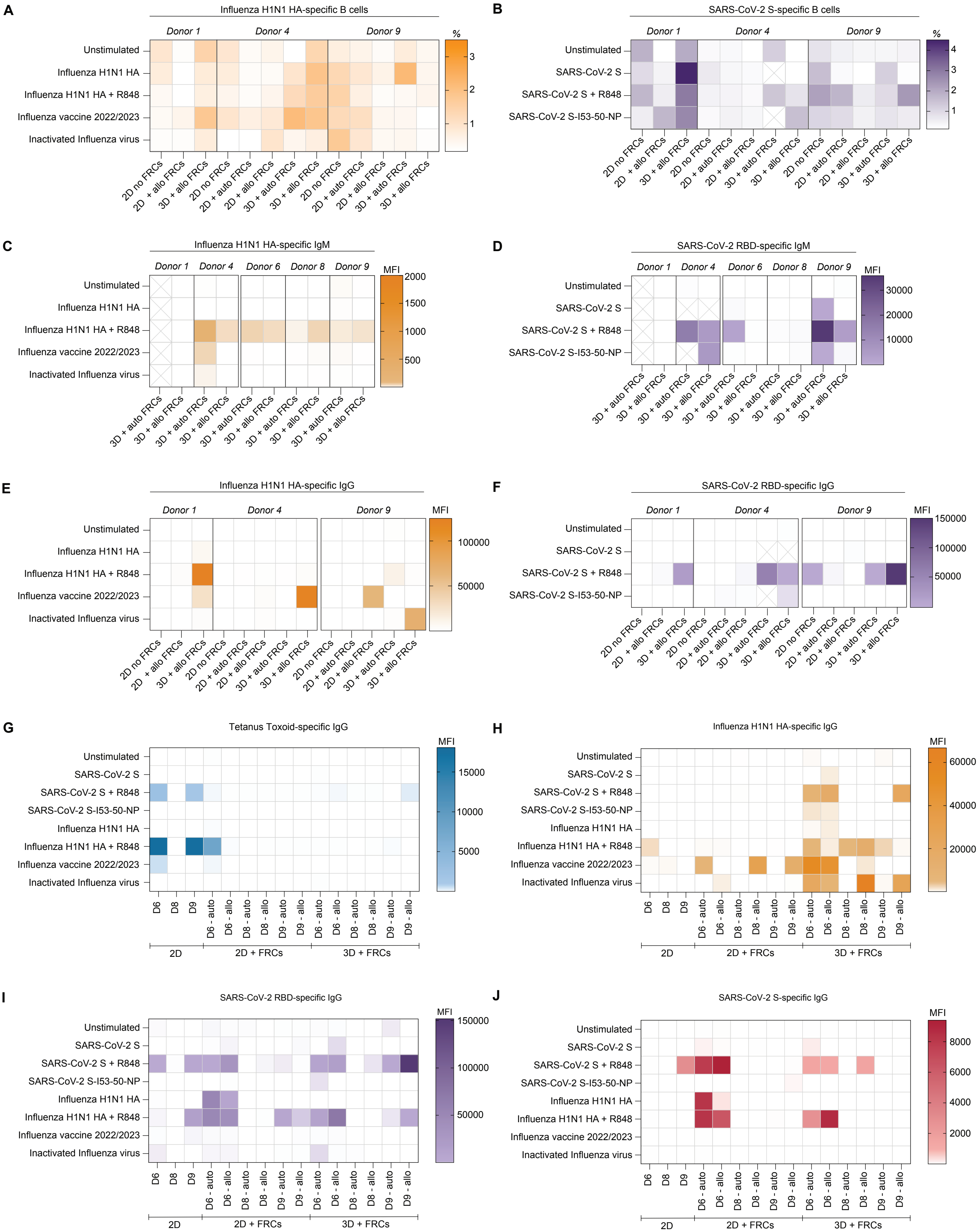

A
Influenza H1N1 HA-specific B cells
Donor 1
Donor 4
Donor 9
%
Unstimulated
Influenza H1N1 HA
Influenza H1N1 HA + R848
Influenza vaccine 2022/2023
Inactivated Influenza virus
2D no FRCs
2D + allo FRCs
3D + allo FRCs
2D no FRCs
2D + auto FRCs
2D + allo FRCs
3D + auto FRCs
3D + allo FRCs
2D no FRCs
2D + auto FRCs
2D + allo FRCs
3D + auto FRCs
3D + allo FRCs
3
2
1
B
SARS-CoV-2 S-specific B cells
Donor 1
Donor 4
Donor 9
%
Unstimulated
SARS-CoV-2 S
SARS-CoV-2 S + R848
SARS-CoV-2 S-I53-50-NP
4
3
2
1
C
Influenza H1N1 HA-specific IgM
Donor 1
Donor 4
Donor 6
Donor 8
Donor 9
MFI
2000
1500
1000
500
3D + auto FRCs
3D + allo FRCs
D
SARS-CoV-2 RBD-specific IgM
MFI
30000
20000
10000
E
Influenza H1N1 HA-specific IgG
MFI
100000
50000
F
SARS-CoV-2 RBD-specific IgG
MFI
150000
100000
50000
G
Tetanus Toxoid-specific IgG
MFI
15000
10000
5000
Unstimulated
SARS-CoV-2 S
SARS-CoV-2 S + R848
SARS-CoV-2 S-I53-50-NP
Influenza H1N1 HA
Influenza H1N1 HA + R848
Influenza vaccine 2022/2023
Inactivated Influenza virus
D6
D8
D9
D6 - auto
D6 - allo
D8 - auto
D8 - allo
D9 - auto
D9 - allo
2D
2D + FRCs
3D + FRCs
H
Influenza H1N1 HA-specific IgG
MFI
60000
40000
20000
I
SARS-CoV-2 RBD-specific IgG
MFI
150000
100000
50000
J
SARS-CoV-2 S-specific IgG
MFI
8000
6000
4000
2000

Figure S5. Analysis of influenza- and SARS-CoV-2-specific B cell responses and bystander activation in tonsil co-cultures. Characterization of both influenza H1N1 hemagglutinin (HA)-specific B cell responses and SARS-CoV-2 wild type (WT) spike-specific B cell responses after culture. Percentages of protein-specific cells are analyzed using flow cytometry. Protein-specific antibody responses were quantified using a Luminex assay, simultaneously quantifying protein unspecific bystander activation. **(A)** Percentage (%) of influenza H1N1 HA-specific B cells out of total living CD19$^+$ B cells on day 14 for tonsil donors 1, 4, and 9. The percentage of influenza HA-specific B cells were quantified using flow cytometry in unstimulated cultures, or cultures stimulated with either influenza H1N1 HA-protein (with and without R848), influenza vaccine 2022/2023 (Influvac Tetra 2022/2023) or whole inactivated recombinant A/Netherlands/602/2009 influenza virus. All cultures were performed either in 2D without FRCs, 2D with autologous FRCs, 2D with allogeneic FRCs, 3D with autologous FRCs, or 3D with allogeneic FRCs. **(B)** Percentage (%) of SARS-CoV-2 WT spike-specific B cells out of total living CD19$^+$ B cells on day 14 for tonsil donors 1, 4, and 9. The percentage of spike-specific B cells was quantified using flow cytometry in an unstimulated culture, or cultures stimulated with either SARS-CoV-2 WT spike (with and without R848) or SARS-CoV-2 WT-I53-50 NPs. All cultures were performed either in 2D without FRCs, 2D with autologous FRCs, 2D with allogeneic FRCs, 3D with autologous FRCs, or 3D with allogeneic FRCs. **(C)** Influenza H1N1 HA-specific IgM production (mean fluorescence intensity (MFI minus blank)) in the supernatants of the 2D and 3D (co-)cultures for tonsil donor 1, 4, and 9, for all culture conditions and antigen stimulations tested. **(D)** SARS-CoV-2 WT receptor-binding domain (RBD)-specific IgM production (mean fluorescence intensity (MFI minus blank)) in the supernatants of the 2D and 3D (co-)cultures for tonsil donor 1, 4, and 9, for all culture conditions and antigen stimulations tested. **(E)** Influenza H1N1 HA-specific IgG production (mean fluorescence intensity (MFI minus blank)) in the supernatants of the 2D and 3D (co-)cultures for tonsil donor 1, 4, and 9, for all culture conditions and antigen stimulations tested. **(F)** SARS-CoV-2 WT receptor-binding domain (RBD)-specific IgG production (mean fluorescence intensity (MFI minus blank)) in the supernatants of the 2D and 3D (co-)cultures for tonsil donor 1, 4, and 9, for all culture conditions and antigen stimulations tested. **(G)** Tetanus toxoid-specific IgG production (mean fluorescence intensity (MFI minus blank)) in the supernatants of the 2D and 3D (co-)cultures for tonsil donor 6, 8, and 9, for all culture conditions and antigen stimulations tested. **(H)** Influenza H1N1 HA-specific IgG production (mean fluorescence intensity (MFI minus blank)) in the supernatants of the 2D and 3D (co-)cultures for tonsil donor 6, 8, and 9, for all culture conditions and antigen stimulations tested. **(I)** SARS-CoV-2 WT receptor-binding domain (RBD)-specific IgG production (mean fluorescence intensity (MFI minus blank)) in the supernatants of the 2D and 3D (co-)cultures for tonsil donor 6, 8, and 9, for all culture conditions and antigen stimulations tested. **(J)** SARS-CoV-2 WT spike-specific IgG production (mean fluorescence intensity (MFI minus blank)) in the supernatants of the 2D and 3D (co-)cultures for tonsil donor 6, 8, and 9, for all culture conditions and antigen stimulations tested.

Data showing the mean of technical duplicates (n=2) from the donors as indicated (A-F).

Figure S6

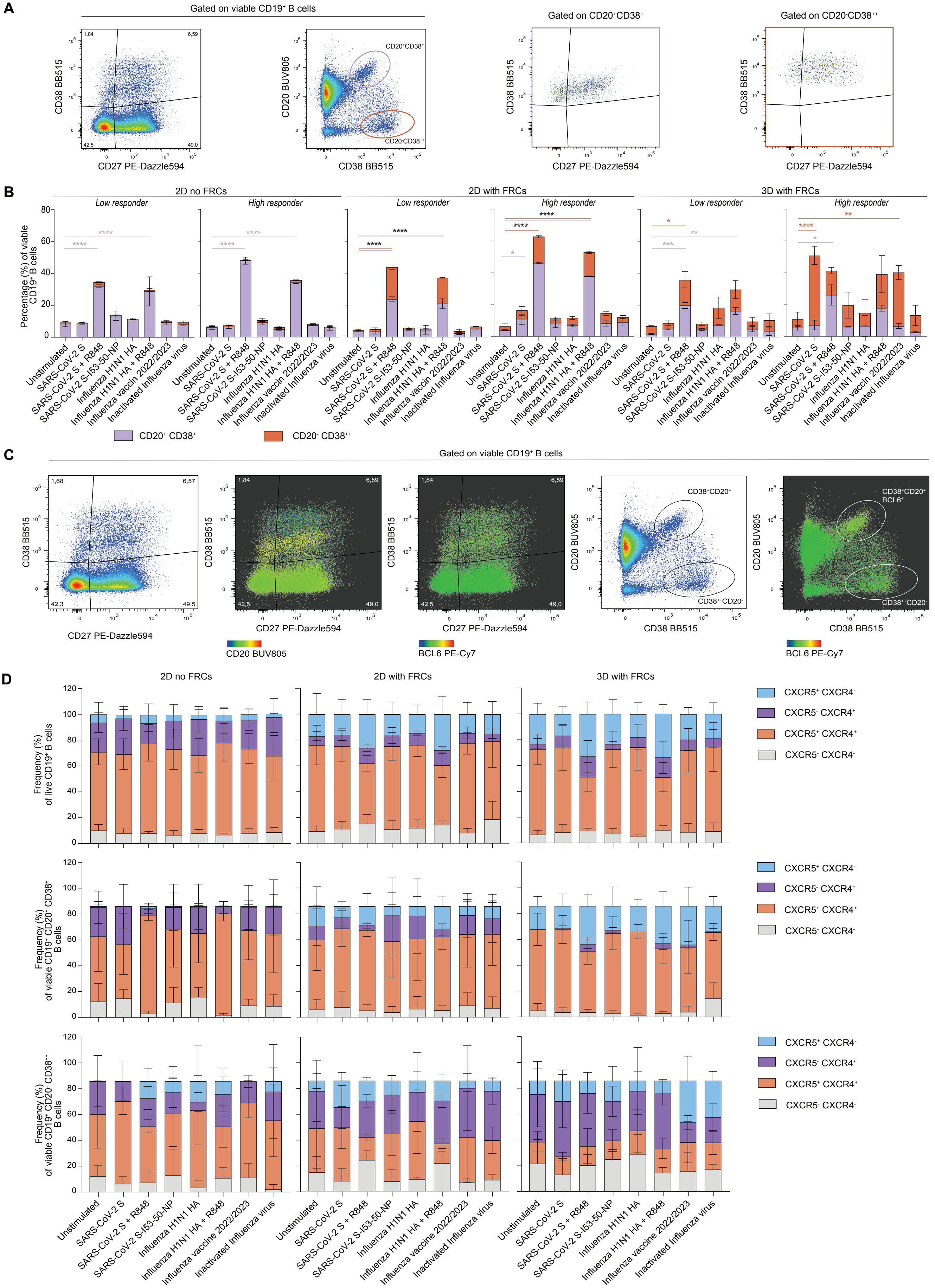
A
Gated on viable CD19+ B cells
Gated on CD20+CD38+
Gated on CD20-CD38++
CD38 BB515
CD27 PE-Dazzle594
CD20 BUV805
CD38 BB515
CD20+CD38+
CD20-CD38++
1,84
6,59
42,5
49,0
B
2D no FRCs
2D with FRCs
3D with FRCs
Low responder
High responder
Percentage (%) of viable CD19+ B cells
Unstimulated
SARS-CoV-2 S
SARS-CoV-2 S + R848
SARS-CoV-2 S-I53-50-NP
Influenza H1N1 HA
Influenza H1N1 HA + R848
Influenza vaccin 2022/2023
Inactivated Influenza virus
CD20+ CD38+
CD20- CD38++
C
Gated on viable CD19+ B cells
1,68
6,57
42,3
49,5
CD38 BB515
CD27 PE-Dazzle594
CD20 BUV805
BCL6 PE-Cy7
CD38+CD20+
CD38++CD20-
CD38+CD20+ BCL6+
D
2D no FRCs
2D with FRCs
3D with FRCs
Frequency (%) of live CD19+ B cells
Frequency (%) of viable CD19+ CD20+ CD38+ B cells
Frequency (%) of viable CD19+ CD20- CD38++ B cells
CXCR5+ CXCR4-
CXCR5- CXCR4+
CXCR5+ CXCR4+
CXCR5- CXCR4-
Unstimulated
SARS-CoV-2 S
SARS-CoV-2 S + R848
SARS-CoV-2 S-I53-50-NP
Influenza H1N1 HA
Influenza H1N1 HA + R848
Influenza vaccine 2022/2023
Inactivated Influenza virus

Figure S6. Gating strategy for germinal center B cells and ASCs and their surface CXCR4/CXCR5 chemokine expression. (A) Representative flow cytometry plot, showing the gating strategy used to identify germinal center (GC) B cells ($CD20^{+}CD38^{+}$) and antibody-secreting cells (ASCs, $CD20^{-}CD38^{++}$) out of total living $CD19^{+}$ B cells. **(B)** Percentage (%) of mature B cell subsets; germinal center (GC) B cells ($CD20^{+}CD38^{+}$) and antibody-secreting cells (ASCs, $CD20^{-}CD38^{++}$), out of total living $CD19^{+}$ B cells on day 14, comparing 2D without FRCs, 2D with autologous FRCs and 3D with autologous FRCs for both the low and high responsive tonsil donor. **(C)** Flow cytometry plots showing the expression of CD20 and BCL6 in relation to CD38 and CD27, or the expression of BCL6 in relation to CD38 and CD20, gated on viable $CD19^{+}$ B cells. **(D)** Surface CXCR4 and CXCR5 chemokine expression of B cells cultured in 2D without FRCs, 2D with autologous FRCs, or 3D with autologous FRCs, either left unstimulated or stimulated with SARS-CoV-2 WT spike protein (with and without R848), SARS-CoV-2 WT-I53-50 NP, influenza H1N1 HA protein (with and without R848), influenza vaccine 2022/2023 (Influvac Tetra 2022/2023) or whole inactivated recombinant A/Netherlands/602/2009 influenza virus. Top row: total living $CD19^{+}$ B cells, middle row: total living $CD19^{+}CD20^{+}CD38^{+}$ B cells (GC-like B cells), bottom row: total living $CD19^{+}CD20^{-}CD38^{++}$ B cells (ASCs).

Data in (B) and (D) showing the mean ± SD (n=5 tonsil donors). $*=p<0.05$, $**=p<0.01$, $***=p<0.001$, $****=p<0.0001$, statistical significance is displayed in the color of the corresponding analyzed experimental group.

Figure S7

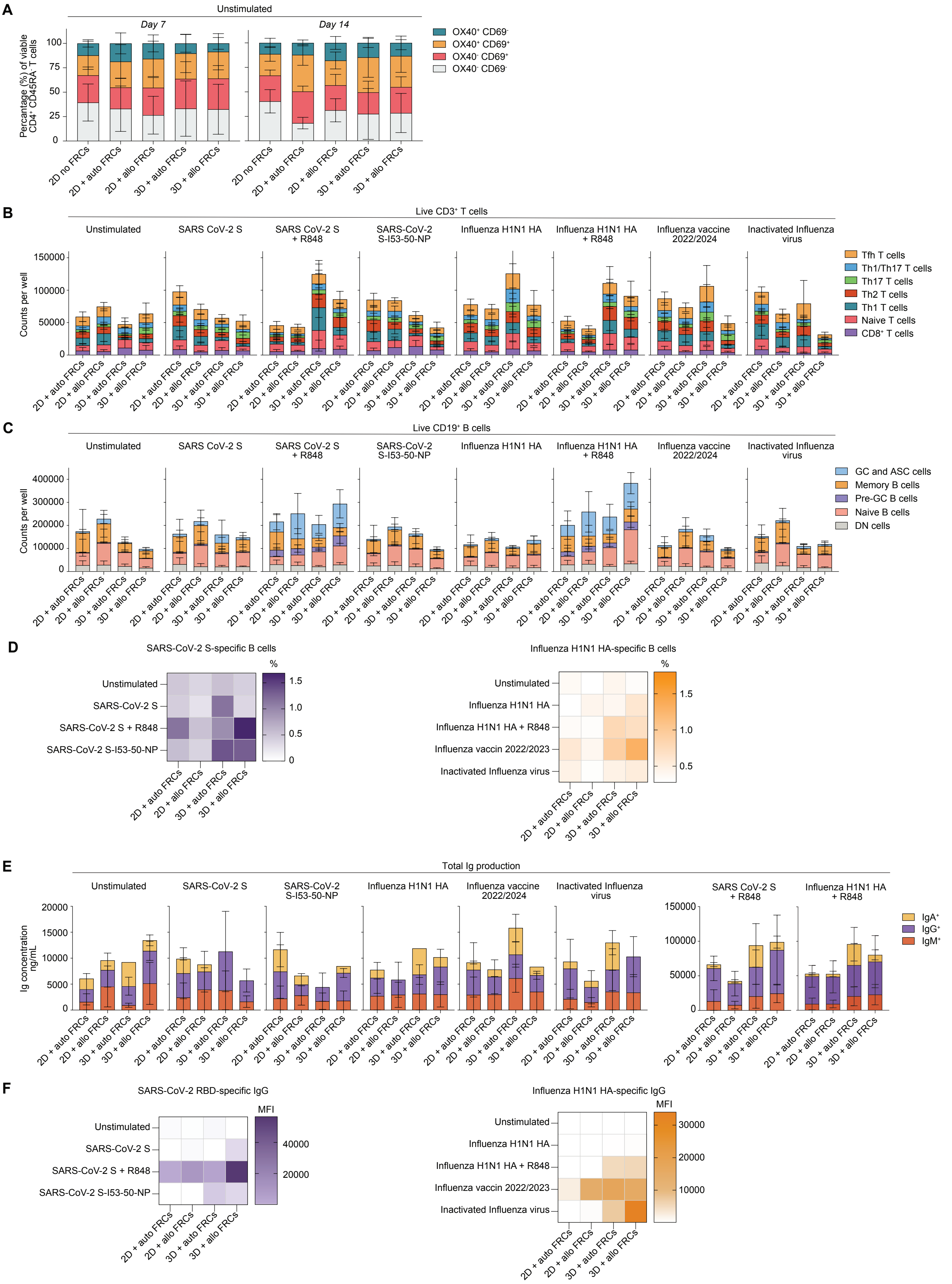
A
Unstimulated
Day 7
Day 14
Percentage (%) of viable CD4⁺ CD45RA⁻ T cells
2D no FRCs
2D + auto FRCs
2D + allo FRCs
3D + auto FRCs
3D + allo FRCs
OX40⁺ CD69⁻
OX40⁺ CD69⁺
OX40⁻ CD69⁺
OX40⁻ CD69⁻
B
Live CD3⁺ T cells
Unstimulated
SARS CoV-2 S
SARS CoV-2 S + R848
SARS-CoV-2 S-I53-50-NP
Influenza H1N1 HA
Influenza H1N1 HA + R848
Influenza vaccine 2022/2024
Inactivated Influenza virus
Counts per well
Tfh T cells
Th1/Th17 T cells
Th17 T cells
Th2 T cells
Th1 T cells
Naive T cells
CD8⁺ T cells
C
Live CD19⁺ B cells
GC and ASC cells
Memory B cells
Pre-GC B cells
Naive B cells
DN cells
D
SARS-CoV-2 S-specific B cells
Unstimulated
SARS-CoV-2 S
SARS-CoV-2 S + R848
SARS-CoV-2 S-I53-50-NP
Influenza H1N1 HA-specific B cells
Influenza H1N1 HA
Influenza H1N1 HA + R848
Influenza vaccin 2022/2023
Inactivated Influenza virus
%
E
Total Ig production
Ig concentration ng/mL
IgA⁺
IgG⁺
IgM⁺
F
SARS-CoV-2 RBD-specific IgG
Influenza H1N1 HA-specific IgG
MFI

Figure S7. Extended comparison of tonsil cell responses in 2D and 3D cultures with or without autologous or allogeneic FRCs. Shown are 2D cultures with autologous or allogeneic FRCs and 3D cultures with autologous or allogeneic FRCs. **(A)** Expression of activation markers OX40 and CD69 on viable $CD4^{+}CD45RA^{-}$ T cells at day 7 (left) and day 14 (right) in unstimulated conditions. **(B)** Total counts of $CD8^{+}$ T cells, naïve $CD4^{+}$ T cells ($CD45RA^{+}$), Th1 ($CD4^{+}CD45RA^{-}CXCR5^{-}CXCR3^{+}CCR6^{-}$), Th2 ($CD4^{+}CD45RA^{-}CXCR5^{-}CXCR3^{-}CCR6^{-}$), Th17 ($CD4^{+}CD45RA^{-}CXCR5^{-}CXCR3^{-}CCR6^{+}$), Th1/17 ($CD4^{+}CD45RA^{-}CXCR5^{-}CXCR3^{+}CCR6^{+}$), and Tfh ($CD4^{+}CD45RA^{-}CXCR5^{+}$) T cells at day 14. **(C)** Total counts of $CD19^{+}$ B cells, including double-negative (DN; $CD27^{-}CD38^{-}IgD^{-}$), naïve ($CD27^{-}CD38^{-}IgD^{+}$), memory ($CD27^{+}CD38^{-}$), pre-GC ($CD27^{-}CD38^{+}$), and GC/antibody-secreting cells (GC and ASC; $CD27^{+}CD38^{+}$) after 14 days of culture, either unstimulated or stimulated with the indicated antigens. **(D)** Percentage of SARS-CoV-2 WT spike-specific B cells (left) and influenza H1N1 HA-specific B cells (right) within total living $CD19^{+}$ B cells on day 14. **(E)** Total immunoglobulin levels (IgM, IgG, and IgA) in culture supernatants on day 14, quantified in ng/mL by ELISA. **(F)** SARS-CoV-2 WT RBD-specific IgG (left) and influenza H1N1 HA-specific IgG (right) in supernatants on day 14, measured by Luminex; MFI values are background-subtracted.
Data are shown as mean ± SD (n = 5 tonsil donors). *$p < 0.05$, **$p < 0.01$, ***$p < 0.001$, ****$p < 0.0001$; statistical significance is indicated in the color of the corresponding experimental group.
